# Sexual selection explains Rensch's rule of allometry for sexual size dimorphism

**DOI:** 10.1098/rspb.2007.1043

**Published:** 2007-09-18

**Authors:** James Dale, Peter O Dunn, Jordi Figuerola, Terje Lislevand, Tamás Székely, Linda A Whittingham

**Affiliations:** 1 Department of Behavioural Ecology and Evolutionary Genetics, Max Planck Institute for OrnithologyPO Box 1564, 82305 Starnberg (Seewiesen), Germany; 2 Department of Biological Sciences, University of Wisconsin-MilwaukeePO Box 413, Milwaukee, WI 53201, USA; 3 Estación Biológica de Doñana, Consejo Superior de Investigaciones CientíficasApartado 1056, 41013 Sevilla, Spain; 4 Bergen Museum, Department of Natural History, University of BergenMuséplass 3, 5007 Bergen, Norway; 5 Department of Biology and Biochemistry, University of BathBath BA2 7AY, UK

**Keywords:** sexual selection, allometry, Rensch's rule, birds, sexual size dimorphism, mating system

## Abstract

In 1950, Rensch first described that in groups of related species, sexual size dimorphism is more pronounced in larger species. This widespread and fundamental allometric relationship is now commonly referred to as ‘Rensch's rule’. However, despite numerous recent studies, we still do not have a general explanation for this allometry. Here we report that patterns of allometry in over 5300 bird species demonstrate that Rensch's rule is driven by a correlated evolutionary change in females to directional sexual selection on males. First, in detailed multivariate analysis, the strength of sexual selection was, by far, the strongest predictor of allometry. This was found to be the case even after controlling for numerous potential confounding factors, such as overall size, degree of ornamentation, phylogenetic history and the range and degree of size dimorphism. Second, in groups where sexual selection is stronger in females, allometry consistently goes in the opposite direction to Rensch's rule. Taken together, these results provide the first clear solution to the long-standing evolutionary problem of allometry for sexual size dimorphism: sexual selection causes size dimorphism to correlate with species size.

## 1. Introduction

To illustrate the nature of ‘Rensch's rule’ (Rensch 1950), consider allometry for sexual size dimorphism in New World blackbirds (Icterinae). When size dimorphism is standardized as the proportional difference between males and females (i.e. the difference in log-transformed wing lengths), there is a clear positive relationship between size dimorphism and log-transformed male size (figure 1*a*). A more statistically robust method (Abouheif & Fairbairn 1997; Fairbairn 1997) for quantifying this size allometry involves plotting the log-transformed values of male versus female wing length (figure 1*b*). Reduced major axis (RMA) slopes of such plots, which scale independently of which variable is plotted on the *x*-axis (Harvey & Pagel 1991; Bonduriansky 2007), provide a measure of allometry. Slopes above 1.0 indicate positive allometry, and in the case of the Icterinae, a slope of 1.22 indicates that a very strong positive allometry occurs in this group (also see Webster 1992), i.e. the blackbirds exhibit Rensch's rule. Similar patterns of size allometry have been documented in groups as diverse as primates (Clutton-Brock *et al*. 1977), turtles (Berry & Shine 1980), water striders (Andersen 1997), drosophilid flies (Blanckenhorn *et al*. 2007), mites (Colwell 2000), salmonid fishes (Young 2005), grouse (Payne 1984), bustards (Payne 1984; Raihani *et al*. 2006), hummingbirds (Payne 1984; Colwell 2000) and shorebirds (Székely *et al*. 2004).

Despite numerous recent studies on sexual size allometry, we still do not have a general explanation for what causes it (Fairbairn 1997; Blanckenhorn *et al*. 2006). Indeed there are a large number of hypotheses that have been developed to explain how sexual size allometry can arise (Clutton-Brock *et al*. 1977; Smith 1977; Payne 1984; Webster 1992; Abouheif & Fairbairn 1997; Fairbairn 1997), and for simplicity, they can be grouped into three broad categories: (i) evolutionary constraints, (ii) natural selection, and (iii) sexual selection.

- Evolutionary constraints (Clutton-Brock *et al*. 1977; Webster 1992; Fairbairn 1997) argue that size dimorphism evolves as a by-product of each sex responding differently to similar selection pressures on body size. For example, different amounts of genetic variation in males and females could result in sexual size allometry if the sex with more additive genetic variation for body size has a stronger evolutionary response to selection (Leutenegger & Cheverud 1982).
- Natural selection, such as intersexual resource competition, and/or fecundity selection (Clutton-Brock *et al*. 1977; Payne 1984; Webster 1992; Fairbairn 1997), can also give rise to sexual size allometry. For example, if increased body size is associated with a reduction in the amount of interspecific competition (MacArthur 1972), then larger species might be more size dimorphic as the sexes diverge into different ecological niches. Alternatively, if natural selection on fecundity acts primarily on female size (Head 1995), then negative allometry (i.e. inconsistent with Rensch's rule) would be expected as males show a lowered evolutionary response to selection on females (Head 1995; Fairbairn 1997).
- Sexual selection hypotheses predict that directional sexual selection acting more strongly on one sex produces a correlated but weaker evolutionary change in the other sex (Smith 1977; Payne 1984; Webster 1992; Abouheif & Fairbairn 1997; Fairbairn 1997). In the typical situation where male size is under stronger sexual selection, then positive allometry consistent with Rensch's rule is expected (irrespective of whether large or small size is sexually selected). In contrast, in taxa where sexual selection is stronger on female size, negative allometry is expected.

Most researchers generally attribute the probable cause of positive sexual size allometry to sexual selection processes; however, we still do not have a conclusive empirical demonstration that this is the case across a broad range of taxa. The objective of this study is thus to test two critical predictions of the sexual selection hypothesis using a close to complete representation of taxa (subfamilies) within an entire class (Aves). First, prediction 1: groups of related species in which sexual selection on size is stronger in males should demonstrate positive allometry, *independently* of confounding factors such as the overall degree and range of size dimorphism. Second, prediction 2: groups of related species in which sexual selection on size is stronger in females should demonstrate negative allometry, they should go in the opposite direction to Rensch's rule.

## 2. Material and methods

### (a) Wing length measurements

For 5334 avian species, we recorded from the literature (see electronic supplementary material for full bibliography) up to seven different sets of male and female wing length measurements. Each set of wing length measurements comprised the means (or mid-ranges if appropriate) for both males and females measured in a single population. The average number of different sets of measurements per species was 2.5, and we took the means of these for final species values. Wing length measurements are commonly used in size dimorphism studies (Payne 1984; Székely *et al*. 2004; Kruger 2005), are much less diurnally and seasonally variable and more frequently reported than body mass (Kruger 2005), and are generally more representative of overall body size than measures of bill or tarsus measures (Székely *et al*. 2004), which are influenced more strongly by ecological factors and have higher intrinsic measurement error (due to their typically smaller sizes). Across all birds, wing length is strongly correlated with body mass (log-transformed values, *N*=1832 species, *R*^2^=0.89; data from Lislevand *et al*. (2007) and Székely *et al*. (2007)) and is therefore, a highly suitable index of overall body size. Flightless birds are excluded from the analyses.

### (b) Classification of subfamilies

The families of birds were based on the classification provided in del Hoyo *et al*. (1992–2007). These families were subdivided into subfamilies based on the classification hitherto provided in del Hoyo *et al*. (1992–2007; *N*=213) and from adapting the classification in Howard & Moore (1991) for all remaining subfamilies (*N*=66). Although there are continual changes in our understanding of the evolutionary relationships among birds, subfamilial-level classifications are generally robust and monophyletic (Cracraft 1981; Cracraft *et al*. 2004).

### (c) Comparisons of slopes

Subfamily-level allometric slopes for sexual size dimorphism were calculated by regressing log male wing length onto log female wing length (e.g. figure 1*b*). All slopes are type II regression, RMA slopes calculated on species values. Since slopes are ratios (i.e. of the vertical change to the horizontal change), we log transformed them to scale their variances appropriately (for example, this transformation would render RMA slopes of 2 and 0.5, as equally allometric, but in positive and negative directions, respectively).

Based on the adopted bird classification (see above), there are 279 subfamilies within 199 families. Out of these, 249 subfamilies have two or more species each, and we were able to calculate allometric slopes for wing length in 220 of these (in the other 29, we had less than two species values for wing lengths). Variance in slopes is expected to be higher in subfamilies with small ranges in species size, because measuring error and minor stochastic evolutionary changes will overwhelm the true allometric relationship. Therefore, we only analysed slopes in the 182 subfamilies, where there was at least a 20% difference between the largest and smallest species. Variance in slopes (log transformed; see above) was dramatically higher in subfamilies below (s.d.=0.577, *n*=38) than above (s.d.=0.035, *n*=182) this cut-off. On average, each slope was estimated with a total of 29 species (s.d.=31, range=2–160), based on an average of 71% (±27%) species representation. Patterns reported here persist when all (*N*=220) subfamilies are included in the analyses; however, the *R*^2^ are, as expected, much lower.

It is assumed that all species within a group share a more recent common ancestor with each other than they do with any other species in any other group. Therefore, each slope is calculated on a single independent species radiation and can be treated as a statistically independent data point for analysis. Note that since each slope is calculated with species values, and is therefore itself not controlled for phylogenetic interdependence within the group, the statistical significances of individual slopes were not estimated.

### (d) Analysis of independent variables

For most independent variables entered into the models, subfamily values were assigned the mean of species scores (provided in the electronic supplementary material). This approach is routine in comparative studies conducted at higher-order taxonomic levels (e.g. Olson & Owens 2005; Kilner 2006), because it provides an intuitive index of the typical phenotypic state characteristic of a group.

Each independent variable was calculated more specifically as follows: (i) polygamy was quantified for as many species as possible as −1=polyandrous, 0=monogamous and 1=polygynous. Measures of polygamy previously collected from the literature (Dunn *et al*. 2001) were pooled together with additional polygamy scores collected independently (J.D.), and in similar fashion. Subfamily-level polygamy scores (i.e. the mean of species scores) determined independently by two observers (J.D. and P.O.D.) were strongly correlated (*N*=69 subfamilies, *R*^2^=0.750, *p*<0.0001). (ii) Plumage dichromatism was scored subjectively by J.D. from plates in the literature (electronic supplementary material) and was scored as −1=females more colourful, 0=no difference, 1=males slightly more, 2=males clearly more, 3=males much more and 4=males much more with additional ornaments. A comparison of UV–VIS spectrometer-based measurements of sexual dichromatism with visual estimates from Dunn *et al*. (2001) demonstrated that they were highly correlated (*R*^2^=0.53, *N*=978 species, *p*<0.0001; P. Dunn 2007, unpublished data). (iii) Relative testes size (from data in Dunn *et al*. 2001; Lislevand *et al*. 2007) was the residuals of log testes mass regressed onto log male body mass. (iv) Male aerial display (from data in Lislevand *et al*. (2007) and Székely *et al*. (2007)) was scored from descriptions in the literature along a spectrum ranging from 1=ground display to 5=highly acrobatic aerial display. (v) Mean clutch size was from data in Lislevand *et al*. (2007) and Székely *et al*. (2007). (vi) Resource partitioning (from data in Lislevand *et al*. (2007) and Székely *et al*. (2007)) was calculated from 0=complete resource overlap between the sexes to 3=no resource overlap. (vii) Species size=mean of log ((male+female wing length)/2) for all species in the subfamily. (viii) Range of species size=maximum species size−minimum species size. (ix) Size dimorphism=mean species value for log(male wing length)−log(female wing length). (x) Range of dimorphism=maximum species size dimorphism−minimum species size dimorphism. (xi) Number of species=total number of species used to calculate the allometric slope for the subfamily.

To test prediction 1, we present the results of (i) univariate analyses of allometry (as the dependent variable) regressed onto each independent variable separately, (ii) multivariate regression of all independent variables used in a combined model, and (iii) multivariate regression on the independent contrasts, which controls for phylogenetic history. Because data for all the variables were not available for all subfamilies, we conducted the three analyses described above separately on two different datasets. The first dataset provides higher power in terms of the number of subfamilies (*N*=182 subfamilies, seven explanatory variables), and the second dataset provides higher power in terms of the number of explanatory variables (*N*=100 subfamilies, 11 explanatory variables). All analyses assume a lack of systematic sex-specific effects of unmeasured viability and fecundity selection across the subfamilies analysed.

### (e) Phylogenetic analysis

We tested whether independent contrasts (Felsenstein 1985) in slopes were predicted by contrasts in the independent variables in tables 1 and 2. The architecture of our subfamily-level phylogeny (provided in the electronic supplementary material) is adapted from Cracraft *et al*. (2004) who synthesized numerous recent molecular-based studies into a comprehensive family-level phylogeny. A separate analysis of independent contrasts calculated using a different phylogeny (Cockburn's (2006) adaptation of Sibley & Alquist's (1991)) yielded the exact same conclusions (not reported). Independent contrasts (standardized by the root of the summed branch lengths) were calculated with the PDAP:PDTREE (Midford *et al*. 2007) software package for Mesquite (Maddison & Maddison 2004), subfamilies were assumed polytomies within families, degrees of freedom were corrected by subtracting the number of polytomies in the phylogenetic tree (Garland & Diaz-Uriarte 1999) and branch lengths were assigned a value of 1.0.

## 3. Results

### (a) How strong is Rensch's rule in birds?

We used allometric relationships in 182 subfamilies to test how general a phenomenon Rensch's rule is in the birds. Positive allometry occurred in 110 out of 182 (60.4%) subfamilies (Sign test; *p*=0.0061). Mean (geometric) slope in the subfamilies was 1.025 (±0.006 s.e.), which was significantly higher than 1 (*t*-test on log-transformed slopes: *t*_181_=4.14, *p*<0.0001). These results demonstrate that positive allometry occurs at a relatively modest level within the subfamilies. However, there is a great deal of variance in size allometry, and many exceptions to Rensch's rule exist. This variance is useful because it can be used to test predictions about what factors drive Rensch's rule.

### (b) Prediction 1: polygyny predicts positive allometry

To test prediction 1, we first evaluated whether polygamy predicts variance in allometry. Sexual selection on male size is expected to be stronger in polygynous groups than in monogamous or polyandrous groups, because males are under stronger intrasexual competition for mating opportunities (Webster 1992). Univariate analyses suggested that subfamilies that have increased degrees of polygyny also have much stronger positive allometry, as predicted by the sexual selection hypothesis (table 1*a*; figure 2*a*). The above pattern could be confounded, however, by correlations between polygamy and other factors related to allometry such as range of size dimorphism or overall size dimorphism (table 1*a*; figure 2; also see Abouheif & Fairbairn 1997; Fairbairn 1997).

To test whether polygamy *per se* was a good predictor of Rensch's rule, we conducted multivariate analyses (general linear model, GLM) with subfamily slope as the dependent variable. Independent variables entered into the model included seven potentially confounding variables: polygamy; sexual dichromatism; average species size; range of species sizes; average sexual size dimorphism; range of sexual size dimorphism; and number of species sampled. The final model was highly significant (table 1) and predicted 36.6% of variance in slopes, with polygamy being by far the most significant predictor of positive allometry (partial *R*^2^=0.182). The other main predictor of positive allometry was range in sexual size dimorphism (partial *R*^2^=0.082). However, this result is not unexpected because variance in size dimorphism is a requirement for positive allometry to occur. Since it is predicted under both sexual selection and other hypotheses for Rensch's rule, it needs to be controlled for. Finally, there were no significant correlations between allometry and either size dimorphism or dichromatism in the multivariate analysis (table 1*b*; partial *R*^2^=0.011 and 0.015, respectively). This result contrasts with patterns observed in the univariate analysis (table 1*a*; figure 2*c*,*d*), and it suggests that the positive correlations in the univariate analyses were indirect results of size dimorphism and plumage dichromatism each being positively related with degree of polygamy (size dimorphism *R*^2^=0.166, *p*<0.0001; dichromatism *R*^2^=0.159, *p*<0.0001). In sum, groups with high degrees of polygyny demonstrate the greatest degrees of positive allometry, independently of other factors. These results demonstrate conclusively that mating system, a proxy of mating competition, is strongly associated with Rensch's rule.

We conducted a more detailed analysis of intersubfamilial variance in allometry using a smaller number of subfamilies, but with a greater number of potential explanatory variables. In 100 subfamilies, we added four additional variables for which we had data available from previously published studies and that may confound the relationship between allometry and mating system (Dunn *et al*. 2001; Lislevand *et al*. 2007; Székely *et al*. 2007): aerial display agility; resource division between the sexes; relative testes size; and clutch size. The final model in this analysis predicted half the variance in slopes (50.3%; table 2*b*), and strongly corroborated the above analysis: polygamy was again the main predictor of allometry (partial *R*^2^=0.199). In contrast, subfamilies with high degrees of sexual dichromatism (tables 1*b* and 2*b*) and relatively large testes (table 2*b*) did not exhibit stronger positive allometry in the GLMs, suggesting that it is the aspect of sexual selection related to size (i.e. intrasexual competition for mating opportunities), and not coloration or sperm competition, that positively influences allometry. Note that the significant relationship between plumage dichromatism and size allometry (partial *R*^2^=0.060) reported in table 2*b* occurs in the opposite direction predicted: groups with *low* degrees of dichromatism tended to have stronger size allometry (after controlling for other factors).

Interestingly, aerial display agility of males was also a significant predictor of allometry, where subfamilies with more elaborate male aerial displays demonstrated stronger positive allometry (partial *R*^2^=0.066; table 2*b*; also see figure 3*b*). This result provides additional support for the hypothesis that differential sexual selection on size between the sexes drives Rensch's rule. Recall that the sexual selection hypothesis predicts positive allometry when males are under stronger directional selection than females, regardless of whether the males are selected to be larger or smaller. Indeed, in species with elaborate male aerial display, sexual selection is generally argued to be stronger for *smaller* male size because it greatly improves aerodynamic efficiency (Andersson & Norberg 1981; Székely *et al*. 2004; Raihani *et al*. 2006).

So far, we have assumed that each allometric slope is the result of an independent species radiation and can thus be considered an independent data point. Nevertheless, it is still possible that unknown phylogenetically shared factors may drive allometric relationships to similar directions in groups of more closely related subfamilies. Therefore, we also constructed GLMs using independent contrasts (Felsenstein 1985) to control for shared phylogenetic history using a recently published avian family-level phylogeny (Cracraft *et al*. 2004). The phylogenetically controlled analyses yielded the same basic result: polygamy drives Rensch's rule (tables 1*c* and 2*c*; figure 3*a*).

### (c) Prediction 2: polyandry predicts negative allometry

To test prediction 2, we calculated slopes in subfamilies with sex-role reversed mating systems. Out of the 182 subfamilies analysed above, there are four subfamilies which demonstrate clear sex-role reversal (i.e. territorial or male-defence polyandry and male-only parental care occurring in at least 50% of their species): the buttonquails (Turnicidae); the jacanas (Jacanidae); the painted snipe (Rostratulidae); and the phalaropes (Phalaropinae). Collectively, the slopes in these birds are significantly lower than 1 (mean (geometric)=0.890, *t*_3_=5.58, *p*=0.006) and are significantly lower than the slopes in all the other subfamilies (*t*_3,177_=3.69, *p*=0.0003). Four cases of polyandrous subfamilies provide too little power for phylogenetically controlled analysis; however, the previous analyses of independent contrasts (tables 1*c* and 2*c*) demonstrated a minimal phylogenetic effect on patterns of subfamily-level size allometry. This first demonstration of size allometry occurring consistently opposite to Rensch's rule provides critical support to the hypothesis that size allometry is driven by differential size selection between the genders, in this case being atypically stronger in females.

## 4. Discussion

The relative differences in the nature of allometry between groups with different mating strategies are shown in figure 4. The strongest difference occurs between the two groups with the highest expected degrees of differential sexual selection between the sexes. In strongly polygynous subfamilies, characterized by high proportions of species with lekking or defence polygyny mating systems, Rensch's rule is demonstrated clearly and in remarkably similar fashion across a broad range of taxa. In monogamous groups, there are no apparent allometric relationships, despite high variance in sexual dimorphism in size and coloration. In groups with clear gender-role reversal, allometry goes in the opposite direction. These strongly contrasting patterns provide conclusive support for the hypothesis that Rensch's rule is driven by a correlated evolutionary response in one sex to stronger size selection in the other sex.

Why might females (typically) demonstrate a correlated evolutionary response to strong size selection on males? This can occur owing to: (i) *genetic correlation between the sexes*: genes favoured in males, which increase male size, tend to be expressed also in females, but to a reduced degree (Winterbottom 1929; Smith 1977; Lande 1980), (ii) *indirect correlational selection*: larger females offset the increased ecological costs of large size with indirect fitness benefits gained by more competitive (i.e. larger) sons (Clutton-Brock *et al*. 1982; Webster 1992; Fairbairn 1997), and/or (iii) *direct correlational selection*: optimal female size is dependent on average male size (Fairbairn 1997; e.g. if males get too large relative to females then females could get injured during intersexual social interactions such as copulation, thereby favouring increased female size). It will be a considerable challenge to tease out the relative effects of these non-mutually exclusive alternatives.

The first mechanism has been argued unlikely (Clutton-Brock *et al*. 1977; Fairbairn 1997) because it suggests a degree of maladaptedness in female size. In other words, stabilizing ecological selection on female size is expected to favour alleles that break up the correlated response in females. Thus, in time, females are expected to drift back down to their original ecological optimum (Fairbairn 1997; Reeve & Fairbairn 2001). However, allometry will occur before equilibrium is reached, and it is unknown how long it would take natural populations to reach equilibrium. In addition, current evolutionary models do not take into account the possibility that, before equilibrium is reached, selection will favour genetic variation which makes females better suited to their maladapted large size—as these alleles spread, females become better adapted to larger size and the optimal female size for the species will increase. The new equilibrium female size will be larger than female size prior to the start of sexual selection on males. Hence, allometry will persist, but only in groups of species with differential size selection between the sexes, and only for as long as it takes before additional evolutionary changes drown out any allometric size scaling between the sexes.

In our comprehensive analysis of Rensch's rule, using a nearly complete representation of subfamilies within a class (Aves), we have uncovered a key life-history variable that is clearly predictive of size allometry: social mating system. Although our analysis is restricted to birds, our findings are expected to be general. Insects, for example, also show high variance in the degree of allometry occurring between different taxa (Blanckenhorn *et al*. 2007). We predict that the degree of differential size selection operating on the sexes, mediated primarily through intrasexual competition for mating opportunities, will best explain variance in size allometry in all taxa. However, a promising area of future research would be to replicate our methodology in some more fecund taxa such as insects, and test whether natural selection on larger females to lay more eggs can also play a role in explaining variance in sexual size allometry.

## Figures and Tables

**Figure 1:**
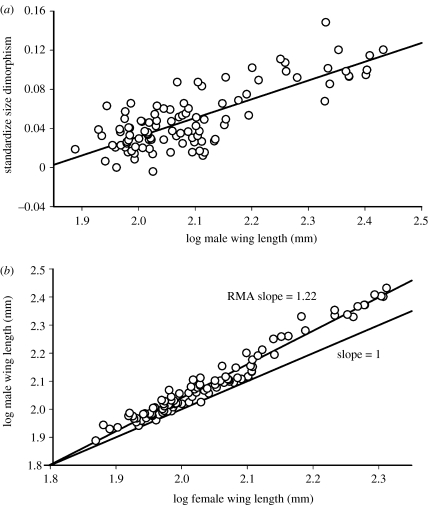
Sexual size allometry in New World blackbirds (Icterinae). (*a*) Standardized size dimorphism (log male wing length−log female wing length) versus male size among 103 out of 105 species in the subfamily. Larger species tend to be proportionally more size dimorphic. (*b*) Log male wing length versus log female wing length plotted from the same sample of species, with the reduced major axis (regression type II) slope calculated. An allometric slope of 1.22 indicates strong positive allometry, i.e. Rensch's rule.

**Figure 2:**
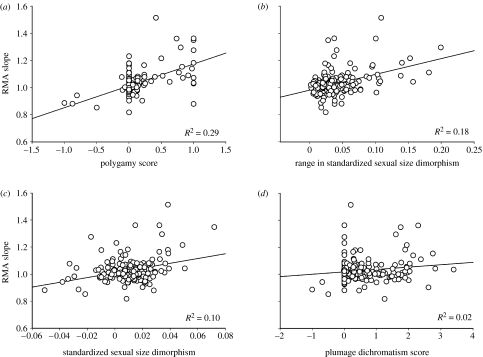
Variance in allometric slopes across 182 subfamilies of birds versus (*a*) degree of polygamy (subfamilies with higher values have proportionally more polygynous species), (*b*) range in sexual size dimorphism, (*c*) mean standardized sexual size dimorphism and (*d*) degree of sexual dichromatism (subfamilies with higher values have more species where males are more colourful than females). Allometric slopes were calculated with RMA models of log male wing length regressed onto log female wing length: higher slopes correspond with stronger positive allometry for sexual size dimorphism, i.e. Rensch's rule. See table 1*a* for the statistics of the regression lines.

**Figure 3:**
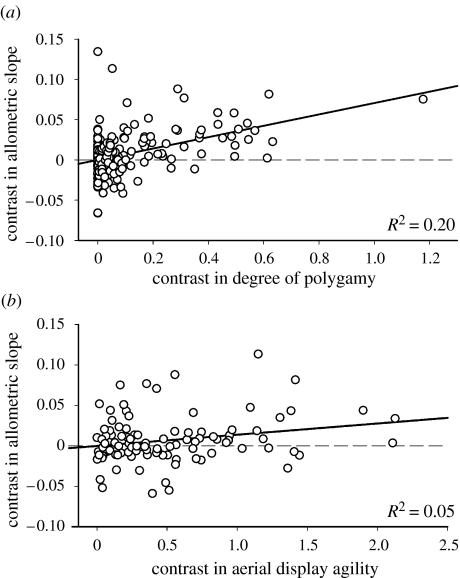
Phylogenetically independent contrasts analysis of allometry for sexual size dimorphism versus (*a*) degree of polygamy, *y*=0.071*x*, *p*<0.0001 (with outlier removed: *y*=0.073*x*, *p*<0.0001, *R*^2^=0.17) and (*b*) aerial display agility, *y*=0.014*x*, *p*=0.001. Contrasts in allometric slopes were calculated on log-transformed values.

**Figure 4:**
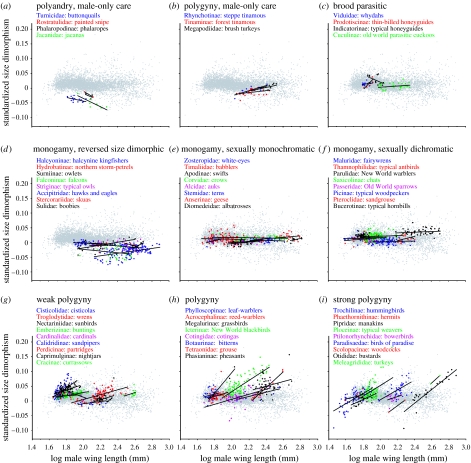
Standardized size dimorphism (log male wing length−log female wing length) versus male size in subfamilies with different mating systems. Polygyny is associated with positive allometry (i.e. slopes >0 in these kinds of plots), and polyandry is associated with negative allometry (i.e. slopes <0). Subfamily names are provided in the order of increasing body size. Grey points comprise the dimorphism versus size relationship for all birds, while coloured points comprise target subfamilies. Representative monogamous subfamilies (*d*–*f*) were selected to cover a broad range of species sizes; however, patterns are similar in other monogamous subfamilies. In (*a*–*c*) and (*g*–*i*), all relevant subfamilies are plotted. Criteria used to categorize subfamilies: (*a*) ≤−0.5 and obligate male-only parental care, (*b*) polygamy >0.10 and some species exhibit obligate male-only parental care, (*c*) obligate interspecific brood parasitism, (*d*) polygamy between −0.06 and 0.06 and size dimorphism <0, (*e*) polygamy between −0.06 and 0.06, and dichromatism between 0 and 0.20, (*f*) polygamy between −0.06 and 0.06, and dichromatism >1.0, (*g*) polygamy between 0.125 and 0.25, (*h*) polygamy between 0.25 and 0.75 and (*i*) polygamy >0.75.

**Table 1:** Linear regression models constructed to predict allometric slopes across 182 subfamilies of birds.

	(*a*) univariate^a^					(*b*) multivariate^b^^,^^c^				(*c*) independent contrasts^b^^,^^d^			
			
independent variable	*β*	*t*	d.f.	*R*^2^	*p*	*β*	*t*	d.f.	*p*	*β*	*t*	d.f.	*p*
polygamy	0.542	8.65	1,180	0.294	<0.0001	0.425	6.28	4,177	<0.0001	0.418	5.96	3,140	<0.0001
dimorphism range	0.424	6.28	1,180	0.180	<0.0001	0.305	3.98	4,177	0.0001	0.236	2.85	3,140	0.005
species size	0.091	1.23	1,180	0.008	0.22	0.146	2.41	4,177	0.017	0.038	0.59	6,137	0.56
size range	0.054	0.73	1,180	0.003	0.47	−0.130	−1.88	4,177	0.06	−0.064	−0.70	5,138	0.48
plumage dichromatism	0.156	2.13	1,180	0.024	0.035	−0.094	−1.39	5,176	0.17	−0.110	−1.53	4,139	0.13
size dimorphism	0.315	4.45	1,180	0.099	<0.0001	0.094	1.38	6,175	0.17	0.020	0.28	7,136	0.78
number of species	0.082	1.10	1,180	0.007	0.27	−0.043	−0.50	7,174	0.62	−0.170	−2.22	3,140	0.028

a Type 1 regression models conducted separately on log-transformed slopes versus each independent variable.

b Non-significant independent terms were removed from successive models in stepwise fashion, starting with the least significant terms. The significance levels for removed terms are the values in the model constructed immediately prior to their removal.

c Final model: *R*^2^=0.366, *F*_4,177_=25.54, *p*<0.0001.

d Multivariate model constructed with independent contrasts (standardized by the root of summed branch lengths), forced through the origin and degrees of freedom corrected by subtracting the number of polytomies (=38) in the phylogenetic tree (Garland & Diaz-Uriarte 1999).

**Table 2:** Linear regression models constructed to predict allometric slopes across 100 subfamilies of birds.

	(*a*) univariate^a^					(*b*) multivariate^b^^,^^c^				(*c*) independent contrasts^b^^,^^d^			
			
independent variable	*β*	*t*	d.f.	*R*^2^	*p*	*β*	*t*	d.f.	*p*	*β*	*t*	d.f.	*p*
polygamy	0.642	8.28	1,98	0.41	<0.0001	0.585	5.52	5,94	<0.0001	0.500	5.15	6,71	<0.0001
plumage dichromatism	0.228	2.32	1,98	0.052	0.023	−0.258	−2.80	5,94	0.006	−0.262	−2.97	6,71	0.004
dimorphism range	0.417	4.54	1,98	0.174	<0.0001	0.171	2.08	5,94	0.040	0.228	2.59	6,71	0.011
display agility	0.233	2.37	1,98	0.054	0.020	0.159	2.02	5,94	0.046	0.280	3.49	6,71	0.0007
size dimorphism	0.418	4.55	1,98	0.175	<0.0001	0.187	1.96	5,94	0.052	0.220	2.55	6,71	0.012
size range	−0.038	−0.379	1,98	0.001	0.71	−0.137	−1.75	6,93	0.08	−0.189	−2.29	6,71	0.024
species size	0.039	0.390	1,98	0.002	0.70	0.127	1.64	7,92	0.11	−0.072	−0.92	7,70	0.36
clutch size	0.180	1.81	1,98	0.032	0.07	0.077	1.01	8,91	0.32	0.034	0.45	9,68	0.65
number of species	0.068	0.672	1,98	0.005	0.50	−0.053	−0.50	9,90	0.62	−0.035	−0.313	10,67	0.76
resource division	0.098	0.979	1,98	0.010	0.33	0.027	0.36	10,89	0.72	−0.009	0.12	11,66	0.91
residual testes size	0.053	0.521	1,98	0.003	0.60	0.021	0.28	11,88	0.78	0.083	1.07	8,69	0.29

a Type 1 regression models conducted separately on log-transformed slopes versus each independent variable.

b Non-significant independent terms were removed from successive models in stepwise fashion, starting with the least significant terms. The significance levels for removed terms are the values in the model constructed immediately prior to their removal.

c Final model: *R*^2^=0.503, *F*_5,94_=19.04, *p*<0.0001.

d Multivariate model constructed with independent contrasts (standardized by the root of summed branch lengths), forced through the origin and degrees of freedom corrected by subtracting the number of polytomies (=22) in the phylogenetic tree (Garland & Diaz-Uriarte 1999).

## Supplementary Material

Supplementary material for Dale *et al* Bibliography, phylogenies, and subfamily data
