# Supplementary material for: Sexual selection explains Rensch's rule of allometry for sexual size dimorphism
Source: Proc Biol Sci. 2007 Sep 18;274(1628):2971–9. doi: 10.1098/rspb.2007.1043 (PMC2211517; doi:10.1098/rspb.2007.1043)
Supplement: Supplementary file 1 [file rspb20071043s05.doc]

ELECTRONIC SUPPLEMENTARY MATERIAL FOR

Dale et al

Sexual selection explains Rensch’s rule of allometry for sexual size dimorphism

Includes:

A. Bibliography for morphological, dichromatism and life history data
B. Phylogeny for GLM 1
C. Phylogeny for GLM 2
D. Data used in the analysis

A.	Bibliography used for wing-length, mating system and plumage dichromatism scores:

1.	Ali, S., and Ripley, S.D. (1978-). Handbook of the birds of India and Pakistan, together with those of Bangladesh, Nepal, Bhutan, and Sri Lanka (in 9 volumes). (Delhi, India: Bombay Natural History Society).

2.	Blake, E.R. (1977). Manual of Neotropical Birds, Volume 1 (Chicago, IL, USA: University of Chicago Press).

3.	Brown, L.H., Urban, E.K., and Newmann, K. eds. (1982-2004). The Birds of Africa (in 7 volumes), Volume 1-7 (London, UK.: Academic Press).

4.	Chantler, P. (2000). Swifts : a guide to the swifts and treeswifts of the world (2nd ed.) (New Haven, USA: Yale University Press).

5.	Cheke, R.A., and Mann, C.F. (2001). Sunbirds : a guide to the sunbirds, flowerpeckers, spiderhunters and sugarbirds of the world (London, UK: Christopher Helm).

6.	Clement, P. (2000). Thrushes (Princeton, NJ, USA: Princeton University Press).

7.	Cramp, S., and Simmons, K.E.L. eds. (1977-1994). Handbook of the birds of Europe, the Middle East and North Africa : the birds of the western Palearctic (in 9 volumes). (Oxford, UK.: Oxford University Press.).

8.	Dale, J. (1992). Mating systems, sexual dimorphism, and phenotypic variation in the bustards (Aves: Otitidae). BSc Thesis thesis, Queen's University, Kingston, ON, Canada.

9.	del Hoyo, J., Elliott, A., and Sargatal, J. eds. (1992-2007). Handbook of the Birds of the World., Volume 1-11 (Barcelona, Spain: Lynx Edicions).

10.	Delacour, J., and Amadon, D. (1973). Currasows and related birds. (New York, NY, USA: American Museum of Natural History).

11.	Dement'ev, G.P., and Gladkov, N.A. (1966-1970). Birds of the Soviet Union Translated from Russian (in 6 volumes) (Jerusalem, Israel: Israel Program for Scientific Translations).

12.	Erritzoe, J., and Erritzoe, H.B. (1998). Pittas of the world : a monograph on the pitta family (Cambridge, UK: Lutterworth).

13.	Ferguson-Lees, J., and Christie, D.A. (2000). Raptors of the World (London, UK: Christopher Helm Publishers).

14.	Ffrench, R. (1991). A guide to the birds of Trinidad and Tobago (Ithaca, NY, USA.: Comstock Pub. Associates).

15.	Fry, H., and Fry, K. (1992). Kingfishers, bee-eaters & rollers : a handbook (Princeton, NJ. USA: Princeton University Press).

16.	Gilliard, E.T. (1969). Birds of paradise and bower birds (London, UK: Weidenfeld & Nicolson).

17.	Godfrey, W.E. (1966). The Birds of Canada (Ottawa, Canada: Queen's Printer).

18.	Harrap, S. (1996). Tits, nuthatches & treecreepers (London, UK: Christopher Helm).

19.	Harris, T. (2000). Shrikes and bush-shrikes : including wood-shrikes, helmet-shrikes, flycatcher-shrikes, philentomas, batises and wattle-eyes (Princeton, NJ, USA.: Princeton University Press).

20.	Hockey, P.A.R., Dean, W.R.J., and Ryan, P.G. (2005). Roberts - Birds of Southern Africa (VIIth edition) (Cape Town, RSA: John Voelcker Bird Book Fund).

21.	Jaramillo, A., and Burke, P. (1999). New world blackbirds : the icterids (Princeton, NJ, USA: Princeton University Press).

22.	Johnsgard, P.A. (1999). The pheasants of the world : biology and natural history (Washington, DC, USA: Smithsonian Institution Press).

23.	Jones, D.N., Dekker, R.W.R.J., and Roselaar, C.S. (1995). The megapodes : Megapodiidae (Oxford, UK: Oxford University Press).

24.	Kemp, A.C. (1995). The hornbills : Bucerotiformes (Oxford, UK: Oxford University Press).

25.	Madge, S., and McGowan, J.K. (2002). A Guide to the Pheasants, Partridges, Quails, Grouse, Guineafowl, Buttonquails and Sandgrouse of the World: Including Buttonquails, Sandgrouse and Allies (London, UK: Christopher Helm Publishers).

26.	Marchant, S., and Higgins, P.J. (1990-2006). Handbook of Australian, New Zealand & Antarctic birds (in 7 volumes) (Melbourne, Australia: Oxford University Press).

27.	Payne, R.B. (1984). Sexual selection, lek and arena behaviour, and sexual size dimorphism in birds. Ornithological Monographs 33, 1-52.

28.	Poole, A., and Gill, F. eds. (2004-). Birds of North America Online (Ithaca, NY, USA.: Cornell Lab of Ornithology.).

29.	Rowley, I., and Russell, E. (1997). Fairy-wrens and grasswrens : Maluridae (Oxford, UK: Oxford University Press).

30.	Short, L.L. (2001). Toucans, barbets and honeyguides : Ramphastidae, Capitonidae, and Indicatoridae (Oxford: Oxford University Press).

31.	Snow, D.W. (1982). The cotingas : bellbirds, umbrellabirds and other species (London, UK: British Museum (Natural History)).

32.	Taylor, P.B. (1998). Rails : a guide to the rails, crakes, gallinules and coots of the world (New Haven, CN, USA: Yale University Press).

33.	Wells, D. (1999). The birds of the Thai-Malay Peninsula : covering Burma and Thailand south of the eleventh parallel, Peninsular Malaysia and Singapore (San Diego, CA, USA: Academic Press).

34.	Wetmore, A. (1965-1984). The birds of the Republic of Panama (in 4 volumes) (Washington DC, USA: Smithsonian Institution).

B.	Phylogeny used for GLM in Table 1:

Tree Description: (('6.1 Tinamidae (Tinaminae): Forest Tinamous':1.0,'6.2 Tinamidae (Rhynchotinae): Steppe Tinamous':1.0):1.0,((('27.2 Anatidae (Anserinae): Swans, Geese and Allies':1.0,'27.3 Anatidae (Anatinae): Ducks, Mergansers and Allies':1.0):1.0,('33 Megapodiidae: Brush Turkeys':1.0,(('34.1 Cracidae (Penelopinae): chachalacas and guans':1.0,'34.2 Cracidae (Cracinae): currassows':1.0):1.0,((('38.1 Phasianidae (Perdicinae): partridges and allies':1.0,'38.2 Phasianidae (Phasianinae): pheasants':1.0,'36 Tetraonidae: Grouse':1.0,'35 Meleagrididae: Turkeys':1.0):1.0,'37 Odontophoridae: New World Quails':1.0):1.0,'39 Numididae: Guineafowls':1.0):1.0):1.0):1.0):1.0,(((('43.1 Gruidae (Gruinae): cranes':1.0,('47 Heliornithidae: Finfoots':1.0,'46 Rallidae: Rails ':1.0):1.0):1.0,'51 Otididae: Bustards':1.0):1.0,('24.1 Threskiornithidae (Threskiornithinae): ibises':1.0,'15 Pelecanidae: Pelecans	':1.0,('20.1 Ardeidae (Ardeinae): day-herons':1.0,'20.3 Ardeidae (Tigrisomatinae): Tiger-herons':1.0,'20.4 Ardeidae (Botaurinae): Bitterns':1.0):1.0,'22 Ciconiidae: Storks':1.0,('16 Sulidae: Boobies ':1.0,'17 Phalacrocoracidae: Cormorants':1.0):1.0,'14 Phaethontidae: Tropicbirds':1.0):1.0,((((('60.1 Charadriidae (Vanellinae): lapwings':1.0,'60.2 Charadriidae (Charadriinae): plovers':1.0):1.0,'57 Recurvirostridae: Stilts and Avocets':1.0):1.0,'58 Burhinidae: Thick-knees ':1.0):1.0,(((('59.1 Glareolidae (Cursoriinae): coursers':1.0,'59.2 Glareolidae (Glareolinae): pratincoles':1.0):1.0,(('65 Stercorariidae: Skuas':1.0,'69 Alcidae: Auks':1.0):1.0,('66 Laridae: Gulls':1.0,'67 Sternidae: Terns':1.0):1.0):1.0):1.0,'42 Turnicidae: Buttonquails':1.0):1.0,((('52 Jacanidae: Jacanas ':1.0,'53 Rostratulidae: Paintedsnipe ':1.0):1.0,'63 Thinocoridae: Seedsnipe':1.0):1.0,('61.1 Scolopacidae (Scolopacinae): woodcocks':1.0,'61.2 Scolopacidae (Gallinagininae): snipes and dowitchers':1.0,'61.3 Scolopacidae (Tringinae): shanks, godwits and curlews':1.0,'61.5 Scolopacidae (Calidridinae): sandpipers':1.0,'61.6 Scolopacidae (Phalaropodinae): phalaropes':1.0):1.0):1.0):1.0):1.0,('25 Phoenicopteridae: Flamingos':1.0,'9 Podicipedidae: Grebes':1.0):1.0,('30 Accipitridae: Hawks and Eagles':1.0,'28 Cathartidae: New World Vultures':1.0):1.0,('32.1 Falconidae (Polyborinae): caracaras and forest falcons':1.0,'32.2 Falconidae (Falconinae): falcons':1.0):1.0):1.0,((('10 Diomedeidae: Albatrosses':1.0,'11 Procellariidae: Petrels & Shearwaters':1.0):1.0,('12.1 Hydrobatidae (Oceanitinae): Southern Storm-Petrels':1.0,'12.2 Hydrobatidae (Hydrobatinae): Northern Storm-Petrels':1.0):1.0):1.0,'8 Gaviidae: Loons ':1.0):1.0):1.0,('76.1 Tytonidae (Tytoninae): Tyto owls':1.0,('77.1 Strigidae (Striginae): typical owls':1.0,'77.2 Strigidae (Surniinae): owlets':1.0,'77.3 Strigidae (Asioninae): eared owls':1.0):1.0):1.0,((('85.1 Trochilidae (Phaethornithinae): hermits':1.0,'85.2 Trochilidae (Trochilinae): typical hummingbirds':1.0):1.0,(('83.1 Apodidae (Cypseloidinae): primitive american swifts':1.0,'83.2 Apodidae (Apodinae): swifts':1.0):1.0,'84 Hemiprocnidae: Crested-Swifts':1.0):1.0):1.0,('81 Nyctibiidae: Potoos':1.0,('82.2.2 Caprimulgidae (Caprimulginae - Caprimulginae): nightjars':1.0,'82.2.1 Caprimulgidae (Caprimulginae - Eurostopodinae): eared nightjars':1.0):1.0):1.0,'80.1 Podargidae (Podarginae): australasian frogmouths':1.0):1.0,'74.3 Musophagidae (Criniferinae): go-away birds':1.0,(('71.1 Columbidae (Columbinae): typical pigeons':1.0,'71.3 Columbidae (Treroninae): fruit-doves':1.0):1.0,'70 Pteroclidae: Sandgrouse':1.0):1.0,(('73.1 Psittacidae (Loriinae): lories':1.0,'73.2 Psittacidae (Psittacinae): parrots':1.0):1.0,'72.2 Cacatuidae (Cacatuinae): white cockatoos':1.0):1.0,('75.5 Cuculidae (Crotophaginae): anis':1.0,'75.6 Cuculidae (Neomorphinae): roadrunners':1.0,'75.3 Cuculidae (Centropodinae): coucals':1.0,('75.2 Cuculidae (Phaenicophaeinae): malkohas and couas':1.0,'75.4 Cuculidae (Coccyzinae): american cuckoos':1.0):1.0,'75.1 Cuculidae (Cuculinae): old world parasitic cuckoos':1.0):1.0,((('99 Bucconidae: Puffbirds':1.0,'98 Galbulidae: Jacamars ':1.0):1.0,((('100.4 Capitonidae (Megalaimatinae): typical asian barbets':1.0,('100.2 Capitonidae (Lybiinae): african arboreal barbets':1.0,'100.1 Capitonidae (Trachyphoninae): african terrestrial barbets':1.0):1.0):1.0,('101 Ramphastidae: Toucans':1.0,('100.5 Capitonidae (Capitoninae): typical american barbets':1.0,'100.6 Capitonidae (Semnornithinae): abberant american barbets':1.0):1.0):1.0):1.0,(('102.1 Indicatoridae (Prodotiscinae): thin-billed honeyguides':1.0,'102.2 Indicatoridae (Indicatorinae): typical honeyguides':1.0):1.0,'103.3 Picidae (Picinae): typical woodpeckers':1.0):1.0):1.0):1.0,(('87.2 Trogonidae (Harpactinae): asian trogons':1.0,'87.3 Trogonidae (Trogoninae): american trogons':1.0):1.0,('97.1 Bucerotidae (Bucerotinae): typical hornbills':1.0,'96.1 Phoeniculidae (Phoeniculinae): typical woodhoopes':1.0):1.0,('91 Meropidae: Bee-eaters':1.0,(('90 Momotidae: Motmots':1.0,('88.3 Alcedinidae (Cerylinae): ceryline kingfishers':1.0,('88.1 Alcedinidae (Halcyonidae): halcyonine kingfishers':1.0,'88.2 Alcedinidae (Alcedininae): alcedinine kingfishers':1.0):1.0):1.0):1.0,'92 Coraciidae: Rollers':1.0):1.0):1.0):1.0,(('185 Ptilonorhynchidae: Bowerbirds':1.0,(('152 Maluridae: Fairywrens':1.0,(('171 Meliphagidae: Honeyeaters':1.0,'154 Epthianuridae: Australian Chats':1.0):1.0,'153 Acanthizidae: Thornbills':1.0):1.0):1.0,('147 Pomatostomidae: Pseudo-babblers':1.0,('148 Orthonychidae: Logrunners':1.0,(((((((('184.2 Paradisaeidae (Paradisaeinae): Birds of Paradise':1.0,('139 Monarchidae: Monarch Flycatchers':1.0,('186 Corvidae: Crows':1.0,'173 Laniidae: True Shrikes':1.0):1.0):1.0):1.0,'180 Corcoracidae: White-winged Chough and Apostlebird':1.0):1.0,'138 Rhipiduridae: Fantails':1.0):1.0,'177 Dicruridae: Drongos':1.0):1.0,(('137 Platysteiridae: Batises and Wattle-eyes':1.0,('175 Prionopidae: Helmetshrikes':1.0,'174 Malaconotidae: Bushshrikes':1.0):1.0):1.0,('183 Cracticidae: Butcherbirds':1.0,'181 Artamidae: Woodswallows':1.0):1.0):1.0):1.0,'172 Oriolidae: Old World Orioles':1.0,'150 Pachycephalidae: Whistlers':1.0,'122 Campephagidae: Cuckooshrikes':1.0,'192.3 Vireonidae [(Vireoninae): typical vireos':1.0,'149 Eupetidae: Rail-babblers and allies':1.0):1.0,'178 Callaeatidae: Wattlebirds':1.0):1.0,('151 Petroicidae: Australasian Robins':1.0,'144 Picathartidae: Rockfowl':1.0,(((((('135.1 Turdidae (Myadestinae): bluebirds and allies':1.0,'135.2 Turdidae (Turdinae): typical thrushes':1.0,'135.3 Turdidae (Saxicolinae): chats':1.0):1.0,'136 Muscicapidae: Old World Flycatchers':1.0):1.0,('133 Mimidae: Mockingbirds and Thrashers':1.0,'187.1 Sturnidae (Sturninae): Starlings':1.0):1.0):1.0,'128 Bombycillidae: Waxwings':1.0):1.0,('159 Sittidae: Nuthatches':1.0,('132 Troglodytidae: Wrens':1.0,'161 Certhiidae: Tree-creepers':1.0):1.0):1.0,(('167 Dicaeidae: Flowerpeckers':1.0,'164 Nectariniidae: Sunbirds':1.0):1.0,(((('189.3 Ploceidae (Plocepasserinae): Sparrow Weavers':1.0,'189.4 Ploceidae (Ploceinae): Weavers':1.0):1.0,'190 Estrildidae: Waxbills':1.0,'191 Viduidae: Indigobirds and Whydahs':1.0):1.0,'134 Prunellidae: Accentors':1.0):1.0,(((('196 Cardinalidae: Cardinals and Allies':1.0,'197 Thraupidae: Tanagers':1.0):1.0,('198.1 Emberizidae [(Emberizinae)]: buntings':1.0,('199.2 Icteridae [(Icterinae)]: typical icterid blackbirds':1.0,'195 Parulidae: New World Warblers':1.0):1.0):1.0):1.0,('188 Passeridae: Old World Sparrows':1.0,('193.2 Fringillidae [(Carduelinae)]: typical finches':1.0,'194 Drepanididae: Hawaiian Honeycreepers':1.0):1.0):1.0):1.0,'121 Motacillidae: Pipits & Wagtails':1.0):1.0):1.0):1.0):1.0,(('157 Paridae: Tits':1.0,'158 Remizidae: Penduline Tits':1.0):1.0,('119 Alaudidae: Larks':1.0,(('120.2 Hirundinidae (Hirundininae): swallows':1.0,'163 Aegithalidae: Long-tailed Tits':1.0):1.0,('142 Cisticolidae: Cisticolas and allies':1.0,('123 Pycnonotidae: Bulbuls':1.0,(('143.1 Megalurinae: grassbirds':1.0,'143.2 Acrocephalinae: reed-warblers and allies':1.0,'143.3 Phylloscopinae: leaf-warblers':1.0,'143.4 Sylviinae: scrub-warblers':1.0):1.0,('169 Zosteropidae: White-eyes':1.0,('145 Timaliidae: Babblers':1.0,'146 Paradoxornithidae: Parrotbills':1.0):1.0):1.0):1.0):1.0):1.0):1.0):1.0):1.0):1.0):1.0):1.0):1.0):1.0):1.0):1.0,(('104.1 Eurylaimidae (Smithornithinae): typical african broadbills':1.0,'106 Pittidae: Pittas':1.0):1.0,(((('113 Cotingidae: Cotingas':1.0,'115.4 Tyrannidae (Tityrinae): becards':1.0):1.0,'114 Pipridae: Manakins':1.0):1.0,('115.3 Tyrannidae (Tyranninae): kingbirds and allies':1.0,'115.1 Tyrannidae (Elaeniinae): tyrannulets and allies':1.0,'115.2 Tyrannidae (Fluvicolinae): pewees and allies':1.0):1.0):1.0,((('108 Dendrocolaptidae: Woodcreepers	':1.0,'107.2 Furnariidae [(Synallaxinae)]: synallaxine ovenbirds':1.0):1.0,'110 Formicariidae: Ground Antbirds':1.0):1.0,'109 Thamnophilidae: Typical Antbirds':1.0):1.0):1.0):1.0):1.0):1.0):1.0):1.0):1.0;

#### C. Phylogeny used for GLM in Table 2

Tree Description: ((('27.2 Anatidae (Anserinae): Swans, Geese and Allies':1.0,'27.3 Anatidae (Anatinae): Ducks, Mergansers and Allies':1.0):1.0,('33 Megapodiidae: Brush Turkeys':1.0,((('38.1 Phasianidae (Perdicinae): partridges and allies':1.0,'38.2 Phasianidae (Phasianinae): pheasants':1.0,'36 Tetraonidae: Grouse':1.0,'35 Meleagrididae: Turkeys':1.0):1.0,'37 Odontophoridae: New World Quails':1.0):1.0,'39 Numididae: Guineafowls':1.0):1.0):1.0):1.0,((('43.1 Gruidae (Gruinae): cranes':1.0,'46 Rallidae: Rails	':1.0):1.0,(('20.1 Ardeidae (Ardeinae): day-herons':1.0,'20.4 Ardeidae (Botaurinae): Bitterns':1.0):1.0,'17 Phalacrocoracidae: Cormorants ':1.0):1.0,((('60.2 Charadriidae (Charadriinae): plovers':1.0,'58 Burhinidae: Thick-knees ':1.0):1.0,((('59.2 Glareolidae (Glareolinae): pratincoles':1.0,(('65 Stercorariidae: Skuas':1.0,'69 Alcidae: Auks':1.0):1.0,('66 Laridae: Gulls':1.0,'67 Sternidae: Terns':1.0):1.0):1.0):1.0,'42 Turnicidae: Buttonquails ':1.0):1.0,((('52 Jacanidae: Jacanas ':1.0,'53 Rostratulidae: Paintedsnipe ':1.0):1.0,'63 Thinocoridae: Seedsnipe':1.0):1.0,('61.1 Scolopacidae (Scolopacinae): woodcocks':1.0,'61.2 Scolopacidae (Gallinagininae): snipes and dowitchers':1.0,'61.3 Scolopacidae (Tringinae): shanks, godwits and curlews':1.0,'61.5 Scolopacidae (Calidridinae): sandpipers':1.0,'61.6 Scolopacidae (Phalaropodinae): phalaropes':1.0):1.0):1.0):1.0):1.0,'9 Podicipedidae: Grebes	':1.0,'30 Accipitridae: Hawks and Eagles':1.0,'32.2 Falconidae (Falconinae): falcons':1.0):1.0,((('10 Diomedeidae: Albatrosses':1.0,'11 Procellariidae: Petrels & Shearwaters':1.0):1.0,('12.1 Hydrobatidae (Oceanitinae): Southern Storm-Petrels':1.0,'12.2 Hydrobatidae (Hydrobatinae): Northern Storm-Petrels':1.0):1.0):1.0,'8 Gaviidae: Loons':1.0):1.0):1.0,('76.1 Tytonidae (Tytoninae): Tyto owls':1.0,('77.1 Strigidae (Striginae): typical owls':1.0,'77.2 Strigidae (Surniinae): owlets':1.0,'77.3 Strigidae (Asioninae): eared owls':1.0):1.0):1.0,(('85.2 Trochilidae (Trochilinae): typical hummingbirds':1.0,'83.2 Apodidae (Apodinae): swifts':1.0):1.0,'82.2.2 Caprimulgidae (Caprimulginae - Caprimulginae): nightjars':1.0,'80.1 Podargidae (Podarginae): australasian frogmouths':1.0):1.0,('71.1 Columbidae (Columbinae): typical pigeons':1.0,'71.3 Columbidae (Treroninae): fruit-doves':1.0):1.0,(('73.1 Psittacidae (Loriinae): lories':1.0,'73.2 Psittacidae (Psittacinae): parrots':1.0):1.0,'72.2 Cacatuidae (Cacatuinae): white cockatoos':1.0):1.0,('75.3 Cuculidae (Centropodinae): coucals':1.0,'75.1 Cuculidae (Cuculinae): old world parasitic cuckoos':1.0):1.0,('103.3 Picidae (Picinae): typical woodpeckers':1.0,(('91 Meropidae: Bee-eaters':1.0,'88.3 Alcedinidae (Cerylinae): ceryline kingfishers':1.0):1.0,'88.1 Alcedinidae (Halcyonidae): halcyonine kingfishers':1.0):1.0,((('152 Maluridae: Fairywrens':1.0,(('171 Meliphagidae: Honeyeaters':1.0,'154 Epthianuridae: Australian Chats':1.0):1.0,'153 Acanthizidae: Thornbills':1.0):1.0):1.0,('147 Pomatostomidae: Pseudo-babblers':1.0,('148 Orthonychidae: Logrunners':1.0,(((('186 Corvidae: Crows':1.0,'173 Laniidae: True Shrikes':1.0):1.0,'177 Dicruridae: Drongos':1.0):1.0,'150 Pachycephalidae: Whistlers':1.0,'149 Eupetidae: Rail-babblers and allies':1.0):1.0,('151 Petroicidae: Australasian Robins':1.0,(((((('135.2 Turdidae (Turdinae): typical thrushes':1.0,'135.3 Turdidae (Saxicolinae): chats':1.0):1.0,'136 Muscicapidae: Old World Flycatchers':1.0):1.0,('133 Mimidae: Mockingbirds and Thrashers':1.0,'187.1 Sturnidae (Sturninae): Starlings':1.0):1.0):1.0,'128 Bombycillidae: Waxwings':1.0):1.0,('159 Sittidae: Nuthatches':1.0,'132 Troglodytidae: Wrens':1.0):1.0,('164 Nectariniidae: Sunbirds':1.0,((('189.4 Ploceidae (Ploceinae): Weavers':1.0,'190 Estrildidae: Waxbills':1.0,'191 Viduidae: Indigobirds and Whydahs':1.0):1.0,'134 Prunellidae: Accentors':1.0):1.0,((('196 Cardinalidae: Cardinals and Allies':1.0,('198.1 Emberizidae [(Emberizinae)]: buntings':1.0,('199.2 Icteridae [(Icterinae)]: typical icterid blackbirds':1.0,'195 Parulidae: New World Warblers':1.0):1.0):1.0):1.0,('188 Passeridae: Old World Sparrows':1.0,'193.2 Fringillidae [(Carduelinae)]: typical finches':1.0):1.0):1.0,'121 Motacillidae: Pipits & Wagtails':1.0):1.0):1.0):1.0):1.0,('157 Paridae: Tits':1.0,('119 Alaudidae: Larks':1.0,('120.2 Hirundinidae (Hirundininae): swallows':1.0,('142 Cisticolidae: Cisticolas and allies':1.0,('123 Pycnonotidae: Bulbuls':1.0,(('143.1 Megalurinae: grassbirds':1.0,'143.2 Acrocephalinae: reed-warblers and allies':1.0,'143.3 Phylloscopinae: leaf-warblers':1.0,'143.4 Sylviinae: scrub-warblers':1.0):1.0,'146 Paradoxornithidae: Parrotbills':1.0):1.0):1.0):1.0):1.0):1.0):1.0):1.0):1.0):1.0):1.0):1.0):1.0,'115.2 Tyrannidae (Fluvicolinae): pewees and allies':1.0):1.0):1.0):1.0):1.0;

**D.	Table of data values used in the analysis.**

Key to the table:

**Taxon:** family#, family name (subfamily name)

**Analysis**: X1s = excluded due to only one species in the subfamily; XSA = excluded due to too small species representation to calculate a slope; XFL = excluded due to flightlessness; XSZ = excluded due to too little species size variation to calculate reliable slope; M1 = included in GLM1 (i.e. table 1); M12 = included in GLM 1 and 2 (i.e. table 1 and 2)

**Spe:** Number of described species in the subfamily

**Sam:** number of species in the subfamily where winglength in both genders was recorded

**Slope:** log 10 transformed Reduced Major Axis slope of log male winglength vs. log female winglength for all species in the subfamily

**Size:** mean species value of ((log male winglength + log female winglength)/2)

**Size_ran** = maximum species value of ((log male winglength + log female winglength)/2) – minimum species value of ((log male winglength + log female winglength)/2)

**Dis_agi** = mean of species scores for display agility

**Res_div** = mean of species score for resource division between the sexes

**Res_tes** = mean of species scores within the subfamily for residuals (calculated across all species of birds) of log(testes mass) regressed onto (log male mass + log female mass)/2

**Clutch** = mean of species scores for clutch size

**Polyg** = mean of species scores for polygamy scores

**Dichrom** = mean of species scores for dichromatism scores

| Taxon | analysis | spe | sam | slope | size | dim | siz_ran | dim_ran | dis_agi | Res_div | res_tes | clutch | polyg | dichrom |
| --- | --- | --- | --- | --- | --- | --- | --- | --- | --- | --- | --- | --- | --- | --- |
| 1 Struthionidae | X1s | 1 | 0 |  |  |  |  |  |  |  |  |  |  |  |
| 2 Rheidae | XSA | 2 | 0 |  |  |  |  |  |  |  |  |  |  |  |
| 3 Casuariidae | XSA | 3 | 0 |  |  |  |  |  |  |  |  |  |  |  |
| 4 Dromaiidae | X1s | 1 | 1 |  |  |  |  |  |  |  |  |  |  |  |
| 5 Apterygidae | XSA | 3 | 0 |  |  |  |  |  |  |  |  |  |  |  |
| 6.1 Tinamidae (Tinaminae) | M1 | 29 | 28 | 0.01237 | 2.23908 | -0.01373 | 0.37902 | 0.04319 |  |  |  |  | 1.00000 | -0.01724 |
| 6.2 Tinamidae (Rhynchotinae) | M1 | 18 | 16 | 0.03721 | 2.23559 | -0.00558 | 0.34963 | 0.04893 |  |  |  |  | 0.91667 | 0.00000 |
| 7 Spheniscidae | XFL | 17 | 15 |  |  |  |  |  |  |  |  |  |  |  |
| 8 Gaviidae | M12 | 4 | 4 | -0.02112 | 2.51339 | 0.01392 | 0.13110 | 0.01028 | 1.00000 | 0.00000 | -0.63019 | 1.94750 | 0.00000 | 0.00000 |
| 9 Podicipedidae | M12 | 20 | 17 | 0.01225 | 2.12998 | 0.01480 | 0.33912 | 0.02929 | 1.14286 | 0.62500 | -0.40462 | 3.85667 | 0.00000 | 0.00000 |
| 10 Diomedeidae | M12 | 14 | 13 | 0.01977 | 2.73933 | 0.01074 | 0.12070 | 0.02139 | 2.22222 | 0.00000 | 0.87980 | 1.00000 | 0.00000 | 0.00000 |
| 11 Procellariidae | M12 | 70 | 54 | 0.01572 | 2.43555 | 0.00366 | 0.46248 | 0.03696 | 2.14286 | 0.07500 | 0.21777 | 1.00000 | 0.00000 | 0.00000 |
| 12.1 Hydrobatidae (Oceanitinae) | M12 | 7 | 5 | 0.01397 | 2.18795 | -0.01190 | 0.11180 | 0.01438 | 3.20000 | 0.00000 | -0.30965 | 1.00000 | 0.00000 | 0.00000 |
| 12.2 Hydrobatidae (Hydrobatinae) | M12 | 13 | 7 | -0.00994 | 2.18418 | -0.00985 | 0.19121 | 0.03216 | 4.00000 | 0.00000 | -1.18643 | 1.00000 | 0.00000 | 0.00000 |
| 13 Pelecanoididae | XSZ | 4 | 4 | 0.02965 |  |  | 0.06716 |  |  |  |  |  |  |  |
| 14 Phaethontidae | M1 | 3 | 3 | 0.03165 | 2.48617 | 0.00331 | 0.09447 | 0.00839 |  |  |  |  | 0.00000 | 0.00000 |
| 15 Pelecanidae | M1 | 7 | 6 | 0.00978 | 2.78737 | 0.03357 | 0.14753 | 0.02217 |  |  |  |  | 0.00000 | 0.00000 |
| 16 Sulidae | M1 | 9 | 9 | 0.07098 | 2.63654 | -0.00984 | 0.11348 | 0.02321 |  |  |  |  | 0.00000 | 0.33333 |
| 17 Phalacrocoracidae | M12 | 39 | 33 | 0.01697 | 2.42716 | 0.01852 | 0.23235 | 0.03574 | 1.13333 | 0.00000 | 0.29007 | 3.03706 | 0.00000 | 0.00000 |
| 18 Anhingidae | XSZ | 2 | 2 | 0.07553 |  |  | 0.02024 |  |  |  |  |  |  |  |
| 19 Fregatidae | XSZ | 5 | 5 | -0.00976 |  |  | 0.06909 |  |  |  |  |  |  |  |
| 20.1 Ardeidae (Ardeinae) | M12 | 34 | 28 | -0.00966 | 2.47243 | 0.01708 | 0.51651 | 0.05194 | 2.42857 | 0.43750 | -0.22741 | 3.52706 | 0.09375 | 0.00000 |
| 20.2 Ardeidae (Nycticoracinae) | XSZ | 8 | 3 | -0.00509 |  |  | 0.04217 |  |  |  |  |  |  |  |
| 20.3 Ardeidae (Tigrisomatinae) | M1 | 5 | 4 | -0.01380 | 2.47666 | 0.01022 | 0.11118 | 0.01087 |  |  |  |  | 0.00000 | 0.00000 |
| 20.4 Ardeidae (Botaurinae) | M12 | 13 | 8 | 0.03321 | 2.32917 | 0.02581 | 0.46880 | 0.04670 | 2.00000 | 1.00000 | -0.40182 | 4.41333 | 0.40000 | 0.53846 |
| 21 Scopidae | X1s | 1 | 1 |  |  |  |  |  |  |  |  |  |  |  |
| 22 Ciconiidae | M1 | 19 | 10 | 0.02601 | 2.72618 | 0.02208 | 0.21171 | 0.04741 |  |  |  |  | 0.00000 | 0.05263 |
| 23 Balaenicipitidae | X1s | 1 | 0 |  |  |  |  |  |  |  |  |  |  |  |
| 24.1 Threskiornithidae (Threskiornithinae) | M1 | 26 | 17 | -0.03172 | 2.51895 | 0.02217 | 0.20227 | 0.04885 |  |  |  |  | 0.00000 | 0.00000 |
| 24.2 Threskiornithidae (Plataleinae) | XSZ | 6 | 3 | 0.11542 |  |  | 0.03058 |  |  |  |  |  |  |  |
| 25 Phoenicopteridae | M1 | 5 | 4 | -0.03405 | 2.58967 | 0.02518 | 0.09318 | 0.02129 |  |  |  |  | 0.00000 | 0.00000 |
| 26 Anhimidae | XSZ | 3 | 3 | 0.01158 |  |  | 0.04605 |  |  |  |  |  |  |  |
| 27.1 Anatidae (Anseranatinae) | X1s | 1 | 1 |  |  |  |  |  |  |  |  |  |  |  |
| 27.2 Anatidae (Anserinae) | M12 | 33 | 30 | 0.00975 | 2.57339 | 0.01755 | 0.52638 | 0.03855 | 1.33333 | 0.80000 | 0.00693 | 6.50474 | 0.00000 | 0.02941 |
| 27.3 Anatidae (Anatinae) | M12 | 113 | 100 | 0.02899 | 2.36766 | 0.02696 | 0.60401 | 0.10486 | 1.71154 | 0.60000 | 0.06329 | 8.41349 | 0.11111 | 1.57522 |
| 28 Cathartidae | M1 | 7 | 6 | 0.04695 | 2.71694 | 0.00432 | 0.29346 | 0.03431 |  |  |  |  | 0.00000 | 0.28571 |
| 29 Pandionidae | X1s | 1 | 1 |  |  |  |  |  |  |  |  |  |  |  |
| 30 Accipitridae | M12 | 237 | 160 | 0.01942 | 2.55242 | -0.02785 | 0.72946 | 0.07461 | 4.39583 | 0.67391 | -0.12534 | 2.37471 | 0.00633 | 0.20693 |
| 31 Sagittariidae | X1s | 1 | 1 |  |  |  |  |  |  |  |  |  |  |  |
| 32.1 Falconidae (Polyborinae) | M1 | 16 | 15 | -0.00054 | 2.46130 | -0.00804 | 0.37019 | 0.02747 |  |  |  |  | 0.00000 | 0.00000 |
| 32.2 Falconidae (Falconinae) | M12 | 45 | 37 | -0.01588 | 2.38775 | -0.03396 | 0.59852 | 0.06249 | 4.35714 | 1.00000 | -0.23229 | 3.44542 | 0.00000 | 0.34444 |
| 33 Megapodiidae | M12 | 19 | 16 | 0.02924 | 2.39646 | 0.00422 | 0.27987 | 0.02335 | 1.00000 | 1.00000 | -0.29921 | 10.14000 | 0.16667 | 0.07143 |
| 34.1 Cracidae (Penelopinae) | M1 | 36 | 34 | 0.01112 | 2.43003 | 0.01836 | 0.33326 | 0.05760 |  |  |  |  | 0.05556 | 0.06944 |
| 34.2 Cracidae (Cracinae) | M1 | 14 | 13 | 0.01656 | 2.56265 | 0.02588 | 0.13405 | 0.04121 |  |  |  |  | 0.16071 | 1.00000 |
| 35 Meleagrididae | M12 | 2 | 2 | 0.12981 | 2.60723 | 0.07202 | 0.09379 | 0.02783 | 1.00000 | 1.00000 | 0.60809 | 10.52000 | 1.00000 | 1.75000 |
| 36 Tetraonidae | M12 | 17 | 17 | 0.13387 | 2.33287 | 0.03223 | 0.31317 | 0.10040 | 1.90909 | 0.40000 | -0.15262 | 8.51308 | 0.73529 | 2.29412 |
| 37 Odontophoridae | M12 | 32 | 32 | 0.02339 | 2.10401 | 0.00966 | 0.22452 | 0.04325 | 1.00000 | 1.00000 | -0.00630 | 9.33333 | 0.06250 | 0.76563 |
| 38.1 Phasianidae (Perdicinae) | M12 | 106 | 97 | 0.03398 | 2.17994 | 0.02064 | 0.64335 | 0.08635 | 1.12500 | 0.68750 | 0.78497 | 6.80000 | 0.13333 | 0.75000 |
| 38.2 Phasianidae (Phasianinae) | M12 | 49 | 48 | 0.01792 | 2.38052 | 0.05068 | 0.44285 | 0.15722 | 1.33333 | 1.25000 | 0.68603 | 7.66667 | 0.62903 | 2.82653 |
| 39 Numididae | M12 | 6 | 6 | -0.02234 | 2.37812 | 0.01603 | 0.16100 | 0.01410 | 1.00000 | 0.00000 | 0.14512 | 7.33333 | 0.10000 | 0.00000 |
| 40 Opisthocomidae | X1s | 1 | 1 |  |  |  |  |  |  |  |  |  |  |  |
| 41 Mesitornithidae | XSA | 3 | 0 |  |  |  |  |  |  |  |  |  |  |  |
| 42 Turnicidae | M12 | 16 | 15 | -0.02595 | 1.93917 | -0.03805 | 0.21864 | 0.02334 | 1.00000 | 0.62500 | -0.00718 | 3.44100 | -0.80556 | -0.68750 |
| 43.1 Gruidae (Balearicinae) | XSA | 2 | 1 |  |  |  |  |  |  |  |  |  |  |  |
| 43.1 Gruidae (Balearicinae) | M12 | 13 | 9 | 0.00224 | 2.75715 | 0.02393 | 0.15563 | 0.03300 | 1.50000 | 0.50000 | 0.24525 | 1.91000 | 0.00000 | 0.00000 |
| 44 Aramidae | X1s | 1 | 1 |  |  |  |  |  |  |  |  |  |  |  |
| 45 Psophiidae | XSZ | 3 | 3 | -0.11517 |  |  | 0.01658 |  |  |  |  |  |  |  |
| 46 Rallidae | M12 | 133 | 121 | 0.01331 | 2.10417 | 0.01252 | 0.73369 | 0.10532 | 1.18421 | 1.27778 | 0.36103 | 5.74095 | 0.02703 | 0.24436 |
| 47 Heliornithidae | M1 | 3 | 3 | 0.07145 | 2.27642 | 0.03839 | 0.24350 | 0.06142 |  |  |  |  | 0.00000 | 0.50000 |
| 48 Rhynochetidae | X1s | 1 | 0 |  |  |  |  |  |  |  |  |  |  |  |
| 49 Eurypygidae | X1s | 1 | 1 |  |  |  |  |  |  |  |  |  |  |  |
| 50 Cariamidae | XSA | 2 | 0 |  |  |  |  |  |  |  |  |  |  |  |
| 51 Otididae | M1 | 25 | 25 | 0.11226 | 2.55694 | 0.03412 | 0.51924 | 0.19929 |  |  |  |  | 0.80000 | 1.60000 |
| 52 Jacanidae | M12 | 8 | 7 | -0.05510 | 2.14378 | -0.05093 | 0.35798 | 0.04834 | 1.00000 | 0.00000 | 0.31288 | 3.72750 | -0.87500 | 0.00000 |
| 53 Rostratulidae | M12 | 2 | 2 | -0.06959 | 2.07791 | -0.02155 | 0.10982 | 0.01756 | 1.00000 | 1.00000 | 0.71536 | 3.88000 | -0.50000 | -0.50000 |
| 54 Dromadidae | X1s | 1 | 1 |  |  |  |  |  |  |  |  |  |  |  |
| 55 Haematopodidae | XSZ | 11 | 9 | -0.03408 |  |  | 0.06253 |  |  |  |  |  |  |  |
| 56 Ibidorhynchidae | X1s | 1 | 1 |  |  |  |  |  |  |  |  |  |  |  |
| 57 Recurvirostridae | M1 | 7 | 7 | -0.02814 | 2.35136 | 0.00930 | 0.10131 | 0.02013 |  |  |  |  | 0.00000 | 0.00000 |
| 58 Burhinidae | M12 | 9 | 7 | 0.06613 | 2.38870 | 0.00403 | 0.14086 | 0.02802 | 4.00000 | 2.00000 | -0.47019 | 1.95200 | 0.00000 | 0.00000 |
| 59.1 Glareolidae (Cursoriinae) | M1 | 9 | 7 | -0.00339 | 2.17591 | 0.00546 | 0.15313 | 0.01818 |  |  |  |  | 0.00000 | 0.00000 |
| 59.2 Glareolidae (Glareolinae) | M12 | 8 | 7 | 0.09008 | 2.24579 | 0.00107 | 0.13907 | 0.03683 | 1.00000 | 0.00000 | -0.33200 | 2.50750 | 0.00000 | 0.00000 |
| 60.1 Charadriidae (Vanellinae) | M1 | 25 | 22 | 0.01234 | 2.29830 | 0.00564 | 0.33015 | 0.02907 |  |  |  |  | 0.00000 | 0.08000 |
| 60.2 Charadriidae (Charadriinae) | M12 | 41 | 37 | -0.00116 | 2.11828 | -0.00038 | 0.30983 | 0.02034 | 2.45455 | 0.95455 | -0.59008 | 3.08955 | -0.05263 | 0.50000 |
| 60.3 Charadriidae (Pluvianellinae) | X1s | 1 | 1 |  |  |  |  |  |  |  |  |  |  |  |
| 61.1 Scolopacidae (Scolopacinae) | M12 | 6 | 2 | 0.10558 | 2.20538 | -0.01737 | 0.16879 | 0.04083 | 4.00000 | 2.00000 | 0.10380 | 3.87000 | 1.00000 | 0.00000 |
| 61.2 Scolopacidae (Gallinagininae) | M12 | 21 | 16 | -0.00984 | 2.14270 | -0.00315 | 0.23825 | 0.02892 | 2.00000 | 1.00000 | -0.46989 | 3.37222 | 0.12500 | 0.04762 |
| 61.3 Scolopacidae (Tringinae) | M12 | 30 | 29 | -0.00785 | 2.26157 | -0.01086 | 0.48471 | 0.02835 | 3.60000 | 0.40000 | -0.32648 | 3.87350 | -0.10000 | 0.25000 |
| 61.4 Scolopacidae (Arenariinae) | XSZ | 2 | 2 | 0.15634 |  |  | 0.01013 |  |  |  |  |  |  |  |
| 61.5 Scolopacidae (Calidridinae) | M12 | 24 | 24 | 0.03945 | 2.08201 | -0.00192 | 0.33007 | 0.10303 | 3.14286 | 0.20000 | 0.48658 | 3.94353 | 0.23684 | 0.31250 |
| 61.6 Scolopacidae (Phalaropodinae) | M12 | 3 | 3 | -0.05243 | 2.09053 | -0.02641 | 0.07785 | 0.01684 | 3.00000 | 0.00000 | 0.14437 | 3.86000 | -1.00000 | -1.00000 |
| 62 Pedionomidae | X1s | 1 | 1 |  |  |  |  |  |  |  |  |  |  |  |
| 63 Thinocoridae | M12 | 4 | 3 | 0.00732 | 2.16990 | -0.00928 | 0.21330 | 0.03148 | 4.00000 | 1.00000 | -0.75673 | 4.00000 | 0.00000 | 0.87500 |
| 64 Chionidae | XSZ | 2 | 2 | -0.04115 |  |  | 0.03817 |  |  |  |  |  |  |  |
| 65 Stercorariidae | M12 | 7 | 6 | -0.02463 | 2.56359 | -0.00718 | 0.13102 | 0.00913 | 1.00000 | 1.00000 | -0.35980 | 1.88000 | 0.00000 | 0.00000 |
| 66 Laridae | M12 | 51 | 42 | 0.01401 | 2.54190 | 0.01625 | 0.32982 | 0.02791 | 1.57143 | 0.37500 | 0.10086 | 2.38581 | 0.04545 | 0.00000 |
| 67 Sternidae | M12 | 44 | 35 | -0.00239 | 2.39991 | 0.00652 | 0.39969 | 0.02176 | 3.61250 | 0.05263 | -0.83543 | 1.63600 | 0.00000 | 0.00000 |
| 68 Rynchopidae | XSZ | 3 | 2 | 0.30028 |  |  | 0.02474 |  |  |  |  |  |  |  |
| 69 Alcidae | M12 | 22 | 20 | 0.00847 | 2.18529 | 0.00287 | 0.36192 | 0.02014 | 1.22222 | 0.00000 | 0.22662 | 1.19625 | 0.00000 | 0.00000 |
| 70 Pteroclidae | M1 | 16 | 16 | 0.02586 | 2.28351 | 0.01222 | 0.19205 | 0.03477 |  |  |  |  | 0.00000 | 2.03125 |
| 71.1 Columbidae (Columbinae) | M12 | 181 | 86 | 0.00268 | 2.19784 | 0.00918 | 0.54071 | 0.03370 | 2.17857 | 0.57895 | 0.12063 | 1.80608 | 0.00000 | 0.49033 |
| 71.2 Columbidae (Otidiphabinae) | X1s | 1 | 0 |  |  |  |  |  |  |  |  |  |  |  |
| 71.3 Columbidae (Treroninae) | M12 | 123 | 23 | -0.00685 | 2.25976 | 0.01009 | 0.35329 | 0.03184 | 2.28571 | 0.71429 | 0.13790 | 1.22600 | 0.00000 | 0.53484 |
| 71.4 Columbidae (Gourinae) | XSA | 3 | 0 |  |  |  |  |  |  |  |  |  |  |  |
| 71.5 Columbidae (Didunculinae) | X1s | 1 | 0 |  |  |  |  |  |  |  |  |  |  |  |
| 72.1 Cacatuidae (Calyptorhynchinae) | XSZ | 6 | 6 | -0.01406 |  |  | 0.06326 |  |  |  |  |  |  |  |
| 72.2 Cacatuidae (Cacatuinae) | M12 | 14 | 7 | 0.02970 | 2.44601 | 0.00745 | 0.12669 | 0.02645 | 1.58333 | 0.35714 | -0.81332 | 2.80000 | 0.00000 | 0.39286 |
| 72.3 Cacatuidae (Nymphicinae) | X1s | 1 | 1 |  |  |  |  |  |  |  |  |  |  |  |
| 73.1 Psittacidae (Loriinae) | M12 | 53 | 6 | 0.01671 | 2.08028 | 0.00710 | 0.15164 | 0.01153 | 1.16667 | 0.30000 | -0.56363 | 3.00000 | 0.00000 | 0.32212 |
| 73.2 Psittacidae (Psittacinae) | M12 | 278 | 89 | 0.00095 | 2.17384 | 0.01109 | 0.71341 | 0.05686 | 1.67442 | 0.64706 | -0.17991 | 4.39018 | 0.04688 | 0.60135 |
| 74.1 Musophagidae (Corythaeolinae) | X1s | 1 | 1 |  |  |  |  |  |  |  |  |  |  |  |
| 74.2 Musophagidae (Musophaginae) | XSZ | 17 | 9 | -0.00994 |  |  | 0.04648 |  |  |  |  |  |  |  |
| 74.3 Musophagidae (Criniferinae) | M1 | 5 | 5 | 0.03862 | 2.35510 | 0.01157 | 0.10271 | 0.02143 |  |  |  |  | 0.00000 | 0.00000 |
| 75.1 Cuculidae (Cuculinae) | M12 | 49 | 35 | 0.00738 | 2.16130 | 0.00724 | 0.36822 | 0.04002 | 2.05000 | 0.85294 | -0.47087 | 2.19875 | 0.23077 | 1.20833 |
| 75.2 Cuculidae (Phaenicophaeinae) | M1 | 25 | 9 | 0.00863 | 2.15152 | -0.00133 | 0.17827 | 0.02069 |  |  |  |  | 0.00000 | 0.00000 |
| 75.3 Cuculidae (Centropodinae) | M12 | 28 | 12 | 0.03226 | 2.26214 | -0.03288 | 0.21194 | 0.06211 | 1.00000 | 1.50000 | 0.67952 | 3.28000 | -0.11111 | 0.00000 |
| 75.4 Cuculidae (Coccyzinae) | M1 | 18 | 5 | -0.01220 | 2.12130 | -0.00186 | 0.14287 | 0.01481 |  |  |  |  | 0.00000 | 0.00000 |
| 75.5 Cuculidae (Crotophaginae) | M1 | 4 | 3 | -0.02863 | 2.19517 | 0.00964 | 0.16763 | 0.01133 |  |  |  |  | 0.00000 | 0.00000 |
| 75.6 Cuculidae (Neomorphinae) | M1 | 10 | 4 | -0.00825 | 2.18159 | 0.01438 | 0.21426 | 0.02518 |  |  |  |  | 0.00000 | 0.00000 |
| 76.1 Tytonidae (Tytoninae) | M12 | 14 | 5 | -0.00709 | 2.47610 | -0.02946 | 0.11985 | 0.05322 | 2.60000 | 1.20000 | -0.52385 | 2.96000 | 0.00000 | 0.50000 |
| 76.2 Tytonidae (Phodilinae) | XSA | 2 | 0 |  |  |  |  |  |  |  |  |  |  |  |
| 77.1 Strigidae (Striginae) | M12 | 117 | 41 | -0.01180 | 2.41069 | -0.01094 | 0.62265 | 0.05232 | 2.12500 | 1.18750 | 0.31185 | 2.89842 | 0.00000 | 0.16667 |
| 77.2 Strigidae (Surniinae) | M12 | 63 | 24 | 0.02073 | 2.18872 | -0.01015 | 0.64349 | 0.05984 | 1.33333 | 1.44444 | -0.03734 | 3.67429 | 0.00000 | 0.14286 |
| 77.3 Strigidae (Asioninae) | M12 | 9 | 4 | 0.04585 | 2.44517 | -0.00151 | 0.12092 | 0.02218 | 4.33333 | 0.66667 | 0.56283 | 3.70000 | 0.00000 | 0.75000 |
| 78 Steatornithidae | X1s | 1 | 1 |  |  |  |  |  |  |  |  |  |  |  |
| 79 Aegothelidae | XSA | 9 | 1 |  |  |  |  |  |  |  |  |  |  |  |
| 80.1 Podargidae (Podarginae) | M12 | 3 | 3 | -0.02001 | 2.38827 | 0.01867 | 0.15126 | 0.01098 | 1.00000 | 1.66667 | -0.56449 | 1.52667 | 0.00000 | 0.83333 |
| 80.2 Podargidae (Batrachostominae) | XSA | 9 | 0 |  |  |  |  |  |  |  |  |  |  |  |
| 81 Nyctibiidae | M1 | 7 | 2 | 0.02983 | 2.49839 | -0.00163 | 0.12399 | 0.00851 |  |  |  |  | 0.00000 | 0.00000 |
| 82.1 Caprimulgidae (Chordeilinae) | XSZ | 10 | 4 | 0.00814 |  |  | 0.05512 |  |  |  |  |  |  |  |
| 82.2 Caprimulgidae (Eurostopodinae) | M1 | 7 | 5 | -0.02704 | 2.34284 | -0.00548 | 0.30607 | 0.02054 |  |  |  |  | 0.00000 | 1.00000 |
| 82.3 Caprimulgidae (Caprimulginae) | M12 | 71 | 32 | 0.02951 | 2.22116 | 0.00746 | 0.21559 | 0.09053 | 2.00000 | 0.60000 | 0.60492 | 1.92750 | 0.13333 | 1.15000 |
| 83.1 Apodidae (Cypseloidinae) | M1 | 13 | 8 | -0.01804 | 2.19312 | 0.00741 | 0.22633 | 0.02042 |  |  |  |  | 0.00000 | 0.00000 |
| 83.2 Apodidae (Apodinae) | M12 | 79 | 48 | 0.01383 | 2.13132 | -0.00025 | 0.37808 | 0.02645 | 3.88889 | 0.25000 | 0.30979 | 2.63190 | 0.00000 | 0.00000 |
| 84 Hemiprocnidae | M1 | 4 | 2 | -0.02002 | 2.15300 | -0.00615 | 0.11567 | 0.00533 |  |  |  |  | 0.00000 | 2.00000 |
| 85.1 Trochilidae (Phaethornithinae) | M1 | 34 | 8 | 0.03018 | 1.75360 | 0.01867 | 0.25933 | 0.03977 |  |  |  |  | 1.00000 | 0.50000 |
| 85.2 Trochilidae (Trochilinae) | M12 | 294 | 74 | 0.07266 | 1.70954 | 0.00941 | 0.38359 | 0.14018 | 3.85714 | 0.75000 | -0.66953 | 1.99733 | 1.00000 | 1.88889 |
| 86.1 Coliidae (Coliinae) | XSZ | 4 | 3 | -0.02812 |  |  | 0.05825 |  |  |  |  |  |  |  |
| 86.2 Coliidae (Urocoliinae) | XSZ | 2 | 2 | 0.15110 |  |  | 0.02005 |  |  |  |  |  |  |  |
| 87.1 Trogonidae (Apalodermatinae) | XSZ | 3 | 3 | 0.05863 |  |  | 0.04231 |  |  |  |  |  |  |  |
| 87.2 Trogonidae (Harpactinae) | M1 | 12 | 6 | 0.02337 | 2.10395 | -0.00267 | 0.14554 | 0.01296 |  |  |  |  | 0.00000 | 1.93750 |
| 87.3 Trogonidae (Trogoninae) | M1 | 24 | 11 | 0.00539 | 2.16272 | 0.00075 | 0.24411 | 0.01867 |  |  |  |  | 0.00000 | 1.96875 |
| 88.1 Alcedinidae (Halcyonidae) | M12 | 59 | 56 | -0.00514 | 2.03771 | -0.00296 | 0.43998 | 0.02783 | 2.37500 | 1.27778 | 0.20877 | 3.41267 | 0.00000 | 0.64407 |
| 88.2 Alcedinidae (Alcedininae) | M1 | 24 | 22 | 0.02033 | 1.81382 | -0.00516 | 0.30801 | 0.02567 |  |  |  |  | 0.00000 | 0.43750 |
| 88.3 Alcedinidae (Cerylinae) | M12 | 9 | 9 | 0.00486 | 2.10666 | -0.00465 | 0.54632 | 0.01223 | 3.00000 | 0.50000 | -0.21190 | 4.50000 | 0.00000 | 1.52778 |
| 89 Todidae | XSA | 5 | 0 |  |  |  |  |  |  |  |  |  |  |  |
| 90 Momotidae | M1 | 10 | 4 | 0.02166 | 2.05148 | 0.00888 | 0.31744 | 0.02500 |  |  |  |  | 0.00000 | 0.00000 |
| 91 Meropidae | M12 | 25 | 25 | 0.01176 | 2.03762 | 0.00759 | 0.29312 | 0.02330 | 2.50000 | 1.00000 | -0.88650 | 3.51444 | 0.00000 | 0.20000 |
| 92 Coraciidae | M1 | 12 | 11 | -0.00172 | 2.24419 | 0.01063 | 0.09930 | 0.02000 |  |  |  |  | 0.00000 | 0.00000 |
| 93 Brachypteraciidae | XSA | 5 | 0 |  |  |  |  |  |  |  |  |  |  |  |
| 94 Leptosomidae | X1s | 1 | 0 |  |  |  |  |  |  |  |  |  |  |  |
| 95 Upupidae | X1s | 1 | 1 |  |  |  |  |  |  |  |  |  |  |  |
| 96.1 Phoeniculidae (Phoeniculinae) | M1 | 5 | 5 | -0.01321 | 2.12098 | 0.02268 | 0.18342 | 0.01214 |  |  |  |  | 0.00000 | 0.45000 |
| 96.2 Phoeniculidae (Rhinopomastinae) | XSZ | 3 | 3 | -0.03495 |  |  | 0.05892 |  |  |  |  |  |  |  |
| 97.1 Bucerotidae (Bucerotinae) | M1 | 52 | 51 | 0.00503 | 2.45348 | 0.03601 | 0.51544 | 0.05385 |  |  |  |  | 0.00000 | 1.80288 |
| 97.2 Bucerotidae (Bucorvinae) | XSZ | 2 | 2 | -3.23631 |  |  | 0.00669 |  |  |  |  |  |  |  |
| 98 Galbulidae | M1 | 18 | 3 | -0.00127 | 1.93846 | -0.00044 | 0.18779 | 0.00227 |  |  |  |  | 0.00000 | 0.66667 |
| 99 Bucconidae | M1 | 35 | 8 | 0.00269 | 1.93099 | -0.00846 | 0.38025 | 0.01495 |  |  |  |  | 0.00000 | 0.08571 |
| 100.1 Capitonidae (Trachyphoninae) | M1 | 5 | 5 | 0.02066 | 1.96548 | 0.00695 | 0.12579 | 0.01456 |  |  |  |  | 0.00000 | 0.60000 |
| 100.2 Capitonidae (Lybiinae) | M1 | 36 | 36 | -0.00164 | 1.88727 | 0.00109 | 0.36604 | 0.02685 |  |  |  |  | 0.00000 | 0.13889 |
| 100.3 Capitonidae (Calorhamphinae) | X1s | 1 | 1 |  |  |  |  |  |  |  |  |  |  |  |
| 100.4 Capitonidae (Megalaimatinae) | M1 | 25 | 25 | 0.00057 | 2.00908 | 0.00012 | 0.26909 | 0.02410 |  |  |  |  | 0.00000 | 0.12000 |
| 100.5 Capitonidae (Capitoninae) | M1 | 13 | 13 | -0.00131 | 1.90062 | 0.00888 | 0.13028 | 0.01718 |  |  |  |  | 0.00000 | 1.76923 |
| 100.6 Capitonidae (Semnornithinae) | M1 | 2 | 2 | 0.00842 | 1.97696 | 0.01030 | 0.07944 | 0.00154 |  |  |  |  | 0.00000 | 0.50000 |
| 101 Ramphastidae | M1 | 34 | 34 | 0.00507 | 2.17389 | 0.01218 | 0.31307 | 0.02947 |  |  |  |  | 0.00000 | 0.86029 |
| 102.1 Indicatoridae (Prodotiscinae) | M1 | 5 | 5 | 0.13417 | 1.87234 | 0.01478 | 0.09196 | 0.03714 |  |  |  |  | 1.00000 | 0.00000 |
| 102.2 Indicatoridae (Indicatorinae) | M1 | 12 | 12 | -0.05604 | 1.94087 | 0.02820 | 0.19657 | 0.03762 |  |  |  |  | 1.00000 | 0.45833 |
| 103.1 Picidae (Jynginae) | XSZ | 2 | 2 | 0.14240 |  |  | 0.04049 |  |  |  |  |  |  |  |
| 103.2 Picidae (Picumninae) | XSZ | 31 | 4 | 0.10818 |  |  | 0.05926 |  |  |  |  |  |  |  |
| 103.3 Picidae (Picinae) | M12 | 183 | 97 | 0.00321 | 2.08648 | 0.00339 | 0.53506 | 0.03957 | 2.45833 | 1.00000 | -0.41012 | 4.07833 | 0.00000 | 1.26639 |
| 104.1 Eurylaimidae (Smithornithinae) | M1 | 3 | 3 | -0.08775 | 1.85694 | 0.00839 | 0.09908 | 0.02410 |  |  |  |  | 0.00000 | 1.25000 |
| 104.2 Eurylaimidae (Calyptomeninae) | XSA | 3 | 0 |  |  |  |  |  |  |  |  |  |  |  |
| 104.3 Eurylaimidae (Eurylaiminae) | XSA | 8 | 0 |  |  |  |  |  |  |  |  |  |  |  |
| 104.4 Eurylaimidae (Pseudocalyptomeninae) | X1s | 1 | 1 |  |  |  |  |  |  |  |  |  |  |  |
| 105 Philepittidae | XSA | 4 | 0 |  |  |  |  |  |  |  |  |  |  |  |
| 106 Pittidae | M1 | 30 | 29 | 0.00512 | 2.06135 | 0.00391 | 0.23872 | 0.02050 |  |  |  |  | 0.00000 | 0.68333 |
| 107.1 Furnariidae (Furnariinae) | XSA | 41 | 0 |  |  |  |  |  |  |  |  |  |  |  |
| 107.2 Furnariidae (Synallaxinae) | M1 | 195 | 24 | 0.02016 | 1.87071 | 0.01901 | 0.27774 | 0.03792 |  |  |  |  | 0.00000 | 0.00513 |
| 108 Dendrocolaptidae | M1 | 52 | 17 | -0.02734 | 2.00164 | 0.02148 | 0.27802 | 0.03731 |  |  |  |  | 0.11765 | 0.00000 |
| 109 Thamnophilidae | M1 | 209 | 31 | 0.00890 | 1.80474 | 0.01092 | 0.36010 | 0.05259 |  |  |  |  | 0.00000 | 1.88876 |
| 110 Formicariidae | M1 | 63 | 8 | 0.00178 | 1.93316 | 0.01139 | 0.22696 | 0.02250 |  |  |  |  | 0.00000 | 0.13095 |
| 111 Conopophagidae | XSA | 8 | 0 |  |  |  |  |  |  |  |  |  |  |  |
| 112 Rhinocryptidae | XSZ | 55 | 3 | -0.50428 |  |  | 0.00815 |  |  |  |  |  |  |  |
| 113 Cotingidae | M1 | 71 | 64 | 0.03603 | 2.08178 | 0.01330 | 0.73095 | 0.13842 |  |  |  |  | 0.52083 | 1.76761 |
| 114 Pipridae | M1 | 58 | 33 | 0.05663 | 1.80523 | 0.00582 | 0.27112 | 0.06861 |  |  |  |  | 0.97436 | 2.13793 |
| 115.1 Tyrannidae (Elaeniinae) | M1 | 203 | 41 | 0.00767 | 1.73959 | 0.02911 | 0.36643 | 0.06470 |  |  |  |  | 0.06436 | 0.06897 |
| 115.2 Tyrannidae (Fluvicolinae) | M12 | 130 | 35 | -0.01415 | 1.84908 | 0.02539 | 0.41890 | 0.03568 | 1.50000 | 0.50000 | -0.17287 | 3.45235 | 0.08333 | 0.33846 |
| 115.3 Tyrannidae (Tyranninae) | M1 | 75 | 32 | -0.01522 | 2.00370 | 0.02221 | 0.23326 | 0.03524 |  |  |  |  | 0.03226 | 0.04000 |
| 115.4 Tyrannidae (Tityrinae) | M1 | 21 | 9 | -0.00911 | 1.92549 | 0.01455 | 0.29684 | 0.02875 |  |  |  |  | 0.00000 | 1.76190 |
| 116 Acanthisittidae | XSZ | 3 | 3 | 0.07004 |  |  | 0.05497 |  |  |  |  |  |  |  |
| 117 Atrichornithidae | XSZ | 2 | 2 | 0.27982 |  |  | 0.06603 |  |  |  |  |  |  |  |
| 118 Menuridae | XSZ | 2 | 2 | 0.58079 |  |  | 0.01229 |  |  |  |  |  |  |  |
| 119 Alaudidae | M12 | 96 | 81 | 0.04461 | 1.95604 | 0.02712 | 0.28188 | 0.05799 | 3.00000 | 1.00000 | 0.05494 | 3.52842 | 0.00000 | 0.29474 |
| 120.1 Hirundinidae (Pseudochelidoninae) | XSA | 2 | 1 |  |  |  |  |  |  |  |  |  |  |  |
| 120.2 Hirundinidae (Hirundininae) | M12 | 81 | 52 | -0.00709 | 2.03637 | 0.00752 | 0.22033 | 0.03805 | 3.00000 | 0.20000 | 0.23937 | 4.58222 | 0.12500 | 0.25000 |
| 121 Motacillidae | M12 | 65 | 49 | 0.02216 | 1.93515 | 0.01825 | 0.19699 | 0.03704 | 3.42857 | 0.91667 | 0.38531 | 4.73000 | 0.00000 | 0.28077 |
| 122 Campephagidae | M1 | 86 | 30 | -0.00003 | 2.03468 | 0.00825 | 0.52811 | 0.03127 |  |  |  |  | 0.00000 | 1.67857 |
| 123 Pycnonotidae | M12 | 138 | 71 | -0.00843 | 1.94633 | 0.02770 | 0.30713 | 0.06877 | 1.50000 | 1.33333 | 0.35299 | 2.30000 | 0.00000 | 0.03442 |
| 124 Chloropseidae | XSZ | 11 | 3 | -0.10648 |  |  | 0.03886 |  |  |  |  |  |  |  |
| 125 Irenidae | XSA | 2 | 1 |  |  |  |  |  |  |  |  |  |  |  |
| 126 Aegithidae | XSZ | 4 | 2 | -0.00684 |  |  | 0.00684 |  |  |  |  |  |  |  |
| 127 Ptiligonatidae | XSA | 4 | 0 |  |  |  |  |  |  |  |  |  |  |  |
| 128 Bombycillidae | M12 | 3 | 3 | -0.01560 | 2.02407 | 0.00337 | 0.08365 | 0.00596 | 1.00000 | 0.00000 | -0.18387 | 4.31000 | 0.00000 | 0.00000 |
| 129 Hypocoliidae | X1s | 1 | 1 |  |  |  |  |  |  |  |  |  |  |  |
| 130 Dulidae | X1s | 1 | 0 |  |  |  |  |  |  |  |  |  |  |  |
| 131 Cinclidae | XSZ | 5 | 3 | -0.02346 |  |  | 0.04890 |  |  |  |  |  |  |  |
| 132 Troglodytidae | M12 | 85 | 10 | 0.00598 | 1.75681 | 0.01722 | 0.27136 | 0.02024 | 2.00000 | 1.50000 | -0.00723 | 5.33444 | 0.14286 | 0.00000 |
| 133 Mimidae | M12 | 34 | 11 | 0.05761 | 2.00698 | 0.00762 | 0.08160 | 0.03238 | 4.00000 | 1.00000 | 0.08360 | 3.42800 | 0.00000 | 0.00000 |
| 134 Prunellidae | M12 | 13 | 10 | 0.00128 | 1.88411 | 0.01747 | 0.17453 | 0.01961 | 2.00000 | 1.00000 | 0.61454 | 4.22800 | -0.06250 | 0.00000 |
| 135.1 Turdidae (Myadestinae) | M1 | 20 | 17 | 0.02699 | 1.99993 | 0.01127 | 0.13633 | 0.02440 |  |  |  |  | 0.00000 | 0.32500 |
| 135.2 Turdidae (Turdinae) | M12 | 133 | 102 | -0.01118 | 2.06507 | 0.01267 | 0.33260 | 0.04058 | 1.85000 | 0.92105 | 0.22823 | 3.39348 | 0.00000 | 0.53383 |
| 135.3 Turdidae (Saxicolinae) | M12 | 183 | 135 | 0.00192 | 1.94261 | 0.02057 | 0.46473 | 0.05716 | 2.88333 | 1.16176 | 0.07163 | 4.60970 | 0.05714 | 1.05055 |
| 136 Muscicapidae | M12 | 116 | 71 | 0.00510 | 1.87915 | 0.01197 | 0.36731 | 0.04597 | 1.92308 | 1.36000 | -0.55121 | 2.90917 | 0.10714 | 1.00431 |
| 137 Platysteiridae | M1 | 30 | 26 | -0.01703 | 1.77581 | 0.00540 | 0.26078 | 0.02880 |  |  |  |  | 0.00000 | 1.68750 |
| 138 Rhipiduridae | M1 | 44 | 8 | 0.03381 | 1.87528 | 0.01597 | 0.24277 | 0.02367 |  |  |  |  | 0.00000 | 0.07955 |
| 139 Monarchidae | M1 | 97 | 30 | 0.00254 | 1.85185 | 0.01572 | 0.32279 | 0.02659 |  |  |  |  | 0.00000 | 1.09794 |
| 140 Regulidae | XSZ | 6 | 4 | -0.02284 |  |  | 0.03526 |  |  |  |  |  |  |  |
| 141 Polioptilidae | XSZ | 17 | 4 | -0.02092 |  |  | 0.05830 |  |  |  |  |  |  |  |
| 142 Cisticolidae | M12 | 145 | 101 | 0.06640 | 1.72375 | 0.03220 | 0.26413 | 0.09569 | 3.20000 | 1.52899 | -0.14674 | 2.90114 | 0.23333 | 0.27414 |
| 143.1 Megalurinae | M12 | 20 | 7 | 0.17999 | 1.82023 | 0.03846 | 0.24814 | 0.10862 | 4.00000 | 1.00000 | 0.17192 | 2.30000 | 0.41667 | 0.20000 |
| 143.2 Acrocephalinae | M12 | 128 | 57 | 0.01955 | 1.81760 | 0.01826 | 0.20910 | 0.10722 | 2.37500 | 1.01190 | -0.17130 | 3.31382 | 0.34211 | 0.05859 |
| 143.3 Phylloscopinae | M12 | 97 | 44 | 0.01291 | 1.75636 | 0.02109 | 0.20139 | 0.06988 | 2.66667 | 1.03571 | 0.14181 | 3.02115 | 0.25000 | 0.00000 |
| 143.4 Sylviinae | M12 | 25 | 21 | 0.01105 | 1.80727 | 0.00739 | 0.21460 | 0.03136 | 3.05882 | 1.25000 | 0.55764 | 4.14222 | 0.12500 | 0.71000 |
| 144 Picathartidae | M1 | 4 | 3 | -0.02104 | 2.10216 | 0.01389 | 0.24170 | 0.01593 |  |  |  |  | 0.00000 | 0.68750 |
| 145 Timaliidae | M1 | 261 | 117 | -0.00551 | 1.91721 | 0.01175 | 0.48911 | 0.07721 |  |  |  |  | 0.00000 | 0.00000 |
| 146 Paradoxornithidae | M12 | 20 | 6 | -0.02217 | 1.87085 | 0.00579 | 0.40769 | 0.03482 | 3.00000 | 0.00000 | 0.18322 | 6.00000 | 0.00000 | 0.12500 |
| 147 Pomatostomidae | M12 | 5 | 4 | -0.00726 | 1.96958 | 0.00579 | 0.14122 | 0.00927 | 1.50000 | 2.00000 | -0.19510 | 3.00000 | 0.00000 | 0.00000 |
| 148 Orthonychidae | M12 | 2 | 2 | 0.01731 | 2.03661 | 0.03926 | 0.14593 | 0.00581 | 1.00000 | 2.00000 | -1.12762 | 1.50000 | 0.00000 | 1.50000 |
| 149 Eupetidae | M12 | 15 | 8 | -0.00153 | 1.95519 | 0.01723 | 0.15712 | 0.01213 | 1.16667 | 1.80000 | -0.08671 | 2.12500 | 0.00000 | 1.06250 |
| 150 Pachycephalidae | M12 | 54 | 14 | -0.00930 | 1.99874 | 0.01279 | 0.22613 | 0.02627 | 1.27778 | 1.61538 | 0.16097 | 2.42857 | 0.00000 | 1.40385 |
| 151 Petroicidae | M12 | 44 | 23 | -0.00571 | 1.90503 | 0.02017 | 0.24246 | 0.04260 | 2.00000 | 1.75000 | 0.10741 | 2.23913 | 0.00000 | 0.80000 |
| 152 Maluridae | M12 | 28 | 28 | -0.00252 | 1.72978 | 0.01418 | 0.29164 | 0.04989 | 1.55556 | 1.55263 | 0.28948 | 2.75000 | 0.00000 | 1.77778 |
| 153 Acanthizidae | M12 | 64 | 46 | -0.00501 | 1.74879 | 0.01637 | 0.27497 | 0.03579 | 1.39844 | 1.26389 | 0.03119 | 2.76667 | 0.00000 | 0.27273 |
| 154 Epthianuridae | M12 | 5 | 5 | 0.02087 | 1.81743 | 0.01182 | 0.09050 | 0.00759 | 2.70000 | 0.60000 | 0.02066 | 2.94200 | 0.00000 | 1.93750 |
| 155 Neosittidae | XSA | 2 | 1 |  |  |  |  |  |  |  |  |  |  |  |
| 156 Climacteridae | XSZ | 7 | 6 | 0.02982 |  |  | 0.03790 |  |  |  |  |  |  |  |
| 157 Paridae | M12 | 52 | 52 | 0.01811 | 1.84825 | 0.01506 | 0.24361 | 0.06449 | 2.20000 | 1.28947 | 0.03141 | 5.43842 | 0.00000 | 0.26471 |
| 158 Remizidae | M1 | 11 | 11 | 0.00245 | 1.72498 | 0.00271 | 0.09049 | 0.01882 |  |  |  |  | 0.10000 | 0.77778 |
| 159 Sittidae | M12 | 24 | 24 | -0.01638 | 1.88870 | 0.01398 | 0.26005 | 0.10135 | 3.00000 | 2.00000 | -0.22120 | 6.72222 | 0.00000 | 0.55556 |
| 160 Tichodromidae | X1s | 1 | 1 |  |  |  |  |  |  |  |  |  |  |  |
| 161 Certhiidae | M1 | 7 | 7 | 0.00591 | 1.84005 | 0.01731 | 0.16614 | 0.00896 |  |  |  |  | 0.00000 | 0.00000 |
| 162 Rhabdornithidae | XSA | 3 | 0 |  |  |  |  |  |  |  |  |  |  |  |
| 163 Aegithalidae | M1 | 8 | 8 | 0.00854 | 1.72784 | 0.01357 | 0.13898 | 0.02132 |  |  |  |  | 0.00000 | 0.06250 |
| 164 Nectariniidae | M12 | 127 | 102 | 0.03281 | 1.77171 | 0.03653 | 0.28007 | 0.05544 | 2.05556 | 1.15789 | -0.05973 | 1.78611 | 0.23864 | 2.03333 |
| 165 Melanocharitidae | XSA | 10 | 0 |  |  |  |  |  |  |  |  |  |  |  |
| 166 Paramythiidae | XSA | 2 | 0 |  |  |  |  |  |  |  |  |  |  |  |
| 167 Dicaeidae | M1 | 44 | 36 | -0.00760 | 1.71701 | 0.02067 | 0.20061 | 0.07710 |  |  |  |  | 0.00000 | 1.45349 |
| 168 Pardalotidae | XSZ | 4 | 4 | 0.02728 |  |  | 0.04184 |  |  |  |  |  |  |  |
| 169 Zosteropidae | M1 | 96 | 20 | 0.02790 | 1.78376 | 0.00556 | 0.17357 | 0.03516 |  |  |  |  | 0.00000 | 0.16667 |
| 170 Promeropidae | XSZ | 2 | 2 | -0.25072 |  |  | 0.01620 |  |  |  |  |  |  |  |
| 171 Meliphagidae | M12 | 177 | 75 | 0.00489 | 1.94724 | 0.02913 | 0.49066 | 0.06214 | 2.72159 | 0.81356 | -0.19144 | 2.23957 | 0.02113 | 0.29412 |
| 172 Oriolidae | M1 | 29 | 13 | -0.01778 | 2.13545 | 0.01002 | 0.11361 | 0.03148 |  |  |  |  | 0.00000 | 1.56250 |
| 173 Laniidae | M12 | 29 | 26 | -0.00377 | 1.98974 | 0.00949 | 0.22211 | 0.04713 | 2.85714 | 1.00000 | 0.05185 | 4.08571 | 0.00000 | 1.12500 |
| 174 Malaconotidae | M1 | 48 | 44 | -0.00852 | 1.95267 | 0.01267 | 0.24979 | 0.04257 |  |  |  |  | 0.00000 | 0.59375 |
| 175 Prionopidae | M1 | 10 | 10 | -0.00103 | 2.07921 | -0.00227 | 0.12478 | 0.02815 |  |  |  |  | 0.00000 | 0.00000 |
| 176 Vangidae | XSA | 14 | 0 |  |  |  |  |  |  |  |  |  |  |  |
| 177 Dicruridae | M12 | 24 | 14 | -0.00006 | 2.13253 | 0.01090 | 0.21138 | 0.01897 | 2.25000 | 0.81250 | -0.12308 | 2.43333 | 0.00000 | 0.00000 |
| 178 Callaeatidae | M1 | 3 | 3 | 0.03387 | 2.15658 | 0.02454 | 0.32634 | 0.02735 |  |  |  |  | 0.00000 | 0.00000 |
| 179 Grallinidae | XSA | 2 | 1 |  |  |  |  |  |  |  |  |  |  |  |
| 180 Corcoracidae | M1 | 2 | 2 | 0.01191 | 2.27239 | -0.00044 | 0.18871 | 0.00517 |  |  |  |  | 0.00000 | 0.00000 |
| 181 Artamidae | M1 | 11 | 7 | -0.04416 | 2.08669 | 0.00705 | 0.11345 | 0.02012 |  |  |  |  | 0.00000 | 0.62500 |
| 182 Pityriaseidae | X1s | 1 | 0 |  |  |  |  |  |  |  |  |  |  |  |
| 183 Cracticidae | M1 | 10 | 8 | 0.00777 | 2.30940 | 0.01789 | 0.27882 | 0.01186 |  |  |  |  | 0.00000 | 0.30000 |
| 184.1 Paradisaeidae (Cnemophilinae) | XSZ | 3 | 3 | 0.14375 |  |  | 0.06836 |  |  |  |  |  |  |  |
| 184.2 Paradisaeidae (Paradisaeinae) | M1 | 41 | 39 | 0.06188 | 2.19569 | 0.03880 | 0.37387 | 0.09482 |  |  |  |  | 0.82051 | 2.75000 |
| 185 Ptilonorhynchidae | M1 | 20 | 18 | 0.02984 | 2.15052 | 0.01045 | 0.15625 | 0.06509 |  |  |  |  | 0.83333 | 0.97500 |
| 186 Corvidae | M12 | 123 | 54 | -0.00194 | 2.31776 | 0.01571 | 0.87397 | 0.02874 | 2.42308 | 1.02885 | 0.14829 | 4.09105 | 0.00000 | 0.00000 |
| 187.1 Sturnidae (Sturninae) | M12 | 112 | 65 | 0.00802 | 2.10125 | 0.01946 | 0.31394 | 0.06589 | 2.15385 | 0.58333 | 0.16504 | 3.55688 | 0.08333 | 0.47500 |
| 187.2 Sturnidae (Buphainae) | XSZ | 2 | 2 | -0.26296 |  |  | 0.01405 |  |  |  |  |  |  |  |
| 188 Passeridae | M12 | 38 | 34 | -0.00089 | 1.89684 | 0.01512 | 0.28895 | 0.02935 | 1.57895 | 0.47727 | 0.42904 | 3.71385 | 0.00000 | 1.19444 |
| 189.1 Ploceidae (Bubalornithinae) | XSZ | 3 | 3 | 0.91471 |  |  | 0.02499 |  |  |  |  |  |  |  |
| 189.2 Ploceidae (Sporopipinae) | XSZ | 2 | 2 | -0.14076 |  |  | 0.06174 |  |  |  |  |  |  |  |
| 189.3 Ploceidae (Plocepasserinae) | M1 | 8 | 8 | 0.00964 | 1.93628 | 0.01260 | 0.26924 | 0.02191 |  |  |  |  | 0.00000 | 0.00000 |
| 189.4 Ploceidae (Ploceinae) | M12 | 104 | 94 | 0.04556 | 1.87992 | 0.03263 | 0.33211 | 0.18132 | 1.43750 | 0.50658 | 0.19087 | 2.37189 | 0.77083 | 2.38043 |
| 190 Estrildidae | M12 | 142 | 96 | 0.00091 | 1.73250 | 0.00591 | 0.22095 | 0.03685 | 1.28571 | 0.82407 | -0.30880 | 4.01667 | 0.11765 | 0.94737 |
| 191 Viduidae | M12 | 18 | 17 | 0.01454 | 1.83342 | 0.01507 | 0.11535 | 0.01965 | 3.08333 | 0.90000 | -0.33182 | 3.26154 | 1.00000 | 3.40000 |
| 192.1 Vireonidae (Cyclarhinae) | XSA | 2 | 1 |  |  |  |  |  |  |  |  |  |  |  |
| 192.2 Vireonidae (Vireolaniinae) | XSA | 4 | 0 |  |  |  |  |  |  |  |  |  |  |  |
| 192.3 Vireonidae (Vireoninae) | M1 | 46 | 14 | -0.01615 | 1.82852 | 0.01106 | 0.17153 | 0.03990 |  |  |  |  | 0.00000 | 0.10000 |
| 193.1 Fringillidae (Fringilla) | XSZ | 3 | 3 | 0.06941 |  |  | 0.05542 |  |  |  |  |  |  |  |
| 193.2 Fringillidae (Carduelinae) | M12 | 137 | 106 | 0.00891 | 1.91380 | 0.01357 | 0.31790 | 0.04850 | 2.90476 | 0.49180 | -0.01703 | 3.68448 | 0.05882 | 2.03125 |
| 194 Drepanididae | M1 | 32 | 27 | -0.01156 | 1.86689 | 0.02228 | 0.25879 | 0.03199 |  |  |  |  | 0.00000 | 1.88889 |
| 195 Parulidae | M12 | 128 | 57 | -0.02410 | 1.80603 | 0.02184 | 0.18635 | 0.02953 | 2.00000 | 1.00000 | 0.06970 | 4.18885 | 0.05882 | 1.30556 |
| 196 Cardinalidae | M12 | 48 | 13 | -0.04442 | 1.91151 | 0.02004 | 0.20885 | 0.03794 | 2.00000 | 0.50000 | 0.17168 | 3.26556 | 0.20455 | 2.61538 |
| 197 Thraupidae | M1 | 256 | 20 | -0.00668 | 1.89125 | 0.01357 | 0.22700 | 0.05548 |  |  |  |  | 0.00000 | 1.48077 |
| 198.1 Emberizidae (Emberizinae) | M12 | 298 | 96 | 0.02147 | 1.85976 | 0.02145 | 0.36935 | 0.03375 | 2.39394 | 0.90854 | 0.26604 | 4.18341 | 0.13265 | 0.52083 |
| 198.2 Emberizidae (Catamblyrhynchinae) | X1s | 1 | 0 |  |  |  |  |  |  |  |  |  |  |  |
| 199.1 Icteridae (dolichonychinae) | X1s | 1 | 1 |  |  |  |  |  |  |  |  |  |  |  |
| 199.2 Icteridae (Icterinae) | M12 | 105 | 104 | 0.08476 | 2.06656 | 0.04905 | 0.49340 | 0.15257 | 2.50000 | 0.50000 | 0.16775 | 3.87833 | 0.50000 | 1.94048 |
